# TRIM44 promotes quiescent multiple myeloma cell occupancy and survival in the osteoblastic niche via HIF-1α stabilization

**DOI:** 10.1038/s41375-018-0222-x

**Published:** 2018-08-08

**Authors:** Zheng Chen, Tsung-Chin Lin, Xiaohong Bi, Guijin Lu, Brian C. Dawson, Roberto Miranda, L. Jeffrey Medeiros, Ian McNiece, Nami McCarty

**Affiliations:** 1 Center for Stem Cell and Regenerative Disease, Brown Foundation Institute of Molecular Medicine for the Prevention of Human Disease, The University of Texas Health Science Center at Houston, Houston, TX USA; 2 Center for Precision Medicine, Brown Foundation Institute of Molecular Medicine for the Prevention of Human Disease, The University of Texas Health Science Center at Houston, Houston, TX USA; 3 Department of Molecular and Human Genetics, Baylor College of Medicine, Houston, TX USA; 4 Department of Hematopathology, The University of Texas, MD Anderson Cancer Center, Houston, TX USA; 5 Department of Stem Cell Transplantation and Cellular Therapy, The University of Texas, MD Anderson Cancer Center, Houston, TX USA

**Keywords:** Myeloma, Cancer stem cells, Myeloma, Cancer stem cells

## Abstract

Despite progress in the treatment of MM, including the use of high-dose chemotherapy and autologous stem cell transplantation, a considerable proportion of patients are refractory to all therapies. This resistance is related to the molecular genetic heterogeneity in MM cells as well as to the contributions from the BM, which is one of the key determinants of treatment outcome. Our previous studies using fluorescent tracers revealed that MM heterogeneity is correlated with the presence of quiescent stem-like cancer cells, which prefer to reside within the osteoblastic niche of the BM. In this report, we identified a novel protein, tripartite motif containing 44 (TRIM44), which is overexpressed in the osteoblastic niche of the BM, enabling MM cells to compete with HSCs for niche support. TRIM44 expression in MM cells promoted cell quiescence but increased bone destruction in xenograft mice, similar to what is observed in MM patients. TRIM44 functions as a deubiquitinase for hypoxia inducible factor-1α (HIF-1α), which stabilizes HIF-1α expression during hypoxia and normoxia. Stabilized HIF-1α stimulates MM cell growth and survival during hypoxia. Our work is the first report to reveal signaling in quiescent MM cells and the functions of TRIM44.

## Introduction

Multiple myeloma (MM) is an incurable B-cell malignancy characterized by the proliferation of plasma cells within the bone marrow (BM) microenvironment. Despite progress in the treatment of MM, including the use of high-dose chemotherapy and autologous stem cell transplantation, a considerable proportion of patients are refractory to all therapies [1]. This resistance is related to the molecular genetic heterogeneity in the MM cells, as well as to the contributions of the BM, which is one of the key determinants of treatment outcome.

Our previous studies using PKH67 fluorescent tracers showed that MM heterogeneity is correlated with the presence of stem-like cancer cells [2]. We isolated MM stem-like cells to near purity on the basis of their ability to retain the lipophilic dye PKH67. As a consequence of their quiescent nature, only MM stem-like cells retain PKH67 in vivo. This study was the first to demonstrate a quiescent MM cell niche and the effects of functional interactions between quiescent MM cells and the microenvironment on MM growth and progression. After cycling in vivo, rare quiescent PKH^+^ cells preferentially reside within osteoblastic (OS) niches rather than in vascular (VS) niches of the BM or spleens. Functional analyses of these cells revealed enhanced colony forming properties in vitro. In addition, these PKH^+^ stem-like cells were highly tumorigenic upon serial transplantation and were resistant to a variety of clinically relevant chemotherapeutic drugs [2].

To delineate the molecular pathways involved in PKH^+^ MM cell functions, we performed gene profiling analyses. Gene profiling analyses of the PKH^+^ and PKH^−^CD138^+^ cells revealed a novel gene called the tripartite motif containing 44 (TRIM44), which was highly upregulated in PKH^+^ cells compared to proliferating cells.

TRIM is a member of the E3 ligase families, which is composed of more than 80 members in human [3]. TRIM family members are involved in many complex cellular functions, including the regulation of immune functions, such as anti-viral responses to autophagy receptor regulators [4, 5], and in cancer development [6]. Except for TRIM44, all TRIM members are E3 ubiquitinases. TRIM44 contains a zinc finger ubiquitin protease domain (UBP) in the N-terminal domains instead of a RING domain, which functions as a deubiquitinase [7]. Even though there is convincing evidence in TRIM44 function related to immune regulation and viral infection, only a handful of publications (total 8) are linked their functions to cancers. For example, TRIM44 is upregulated in head and neck squamous cell carcinoma, lung cancers, prostate cancers and hepatocellular carcinoma with functions varies from promoting migration and invasion to enhancing drug resistance in cancer cells [8–11]. Upregulated TRIM44 is also associated with a poor prognosis in testicular germ cell tumor, esophageal squamous cell carcinoma, and breast cancers [12–16].

A search of the integrated cancer microarray database (Oncomine) further reveals that TRIM44 gene expression is significantly upregulated in MM compared to normal or monoclonal gammopathy of undetermined significance (MGUS, a precursor stage of MM), suggesting that TRIM44 expression may play an oncogenic role, contributing to MM progression. In this study, we report that TRIM44 plays a unique role in controlling MM quiescence and survival in a hypoxic BM niche. TRIM44 upregulation rendered MM cells to be maintained in a quiescent status. TRIM44 over-expressing (TRIM44^OE^) MM cells were equipped to compete with HSCs for niche support, which further increased their localization to the BM. Increased TRIM44^OE^ MM cell engraftment suppressed HSC differentiation into leukocytes. Despite its role in promoting quiescence, TRIM44 upregulation in MM increased bone destruction in xenograft mice, which resembles the human MM pathology. TRIM44-induced MM cell survival within the BM was partly due to hypoxia-inducible factor-1α (HIF-1α) stabilization by TRIM44, which decreases HIF-1α polyubiquitination and degradation by its deubiquitinase activity. Our data unveil novel functions of quiescent MM cells in MM pathology and its relation to MM survival within a hypoxic niche. In addition, our data further support that TRIM44 deubiquitinase plays unique roles in promoting the survival of quiescent MM cells in the BM by stabilizing HIF-1α.

## Results

### MM cells and human HSCs occupy the same niche within the BM

**Fig. 1:**
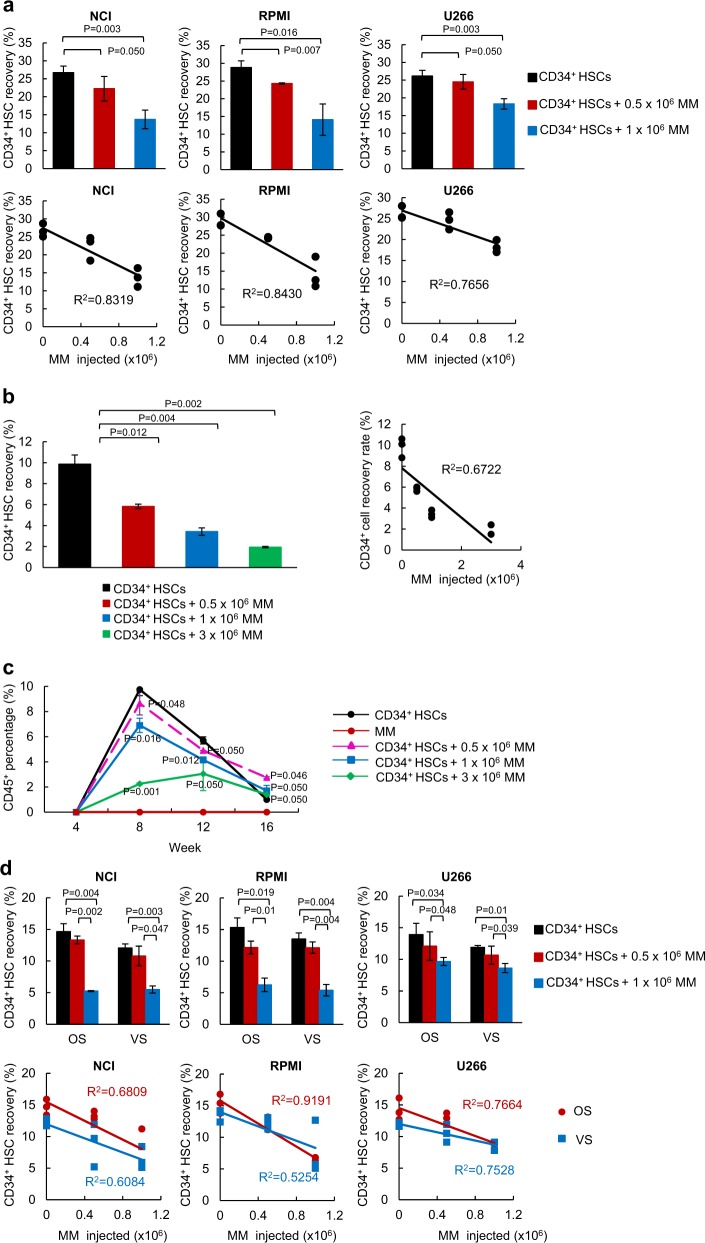

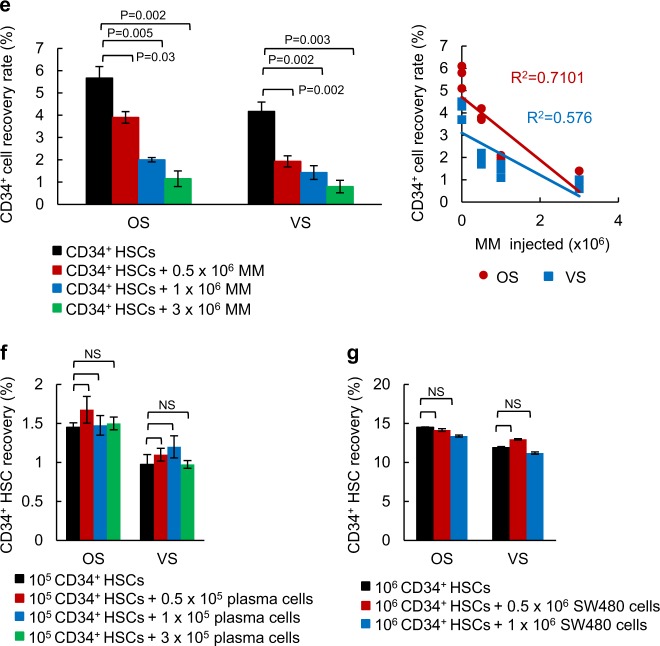
MM cells share a niche with CD34^+^ HSCs in the BM. **a** CD34^+^ HSCs were intravenously (i.v.) transferred into irradiated NOD/SCID mice with different numbers of MM cells (NCI, RPMI, U266, 0, 0.5 × 10^6^ or 1 × 10^6^ cells). After 60 h, the femurs were isolated and CD34^+^ cell recovery was calculated using FACS. The coefficient of determination (*R*^2^) values showed an inverse correlation between HSCs and MM cells. Total 72 mice were used for this experiment. Detailed mice numbers in each experimental group are described in Table S1. **b** CD34^+^ HSCs were i.v. transferred to NSG mice with different numbers of RPMI MM cells (0, 0.5 × 10^6^, 1 × 10^6^ or 3 × 10^6^ cells). After 16 weeks, the femurs were isolated and CD34^+^ cell recovery was calculated using FACS. The *R*^2^ values showed an inverse correlation between the engrafted HSCs and MM cells. Total 24 mice were used for this experiment. Detailed mice numbers in each experimental group are described in Table S2. **c** CD34 HSCs were i.v. transferred to NSG mice with different numbers of RPMI MM cells. Facial blood samples were taken at 4, 8, 12, and 16 weeks post transplantation, and the amount of CD45^+^ leukocytes was analyzed in the blood using FACS. Total 24 mice were used for this experiment. Detailed mice numbers in each experimental group are described in Table S2. **d** MM cells occupy both the osteoblastic (OS) and the vascular (VS) niches in the BM. CD34^+^ HSCs were transferred with different numbers of MM cells into NOD/SCID mice. After engraftment for 60 h, the BM was separated into the OS or the VS niche and CD34^+^ recovery was calculated using FACS. The *R*^2^ analyses showed an inverse correlation between MM cell numbers and HSC recovery rates. A total of 66 mice were used in this experiment. Detailed mice numbers in each experimental group are described in Table S3. **e** CD34^+^ HSCs were i.v. transferred to NSG mice with different numbers of RPMI MM cells (0, 0.5 × 10^6^, 1 × 10^6^, or 3 × 10^6^ cells). After 16 weeks, the BM was separated into the OS and the VS and CD34^+^ cell recovery was calculated using the FACS. The calculated *R*^2^ showed an inverse correlation between the engrafted HSCs and MM cells. The data are represented as the mean ± SD (error bars). The statistical significance was calculated using an unpaired Student’s *t*-test and is represented as a *P*-value. Total 24 mice were used in this experiment. Detailed mice numbers in each experimental group are described in Table S4. **f** Normal plasma cells do not compete with CD34^+^ HSCs for the BM niche. CD34^+^ HSCs were transferred with different numbers of plasma cells into NOD/SCID mice. After engraftment for 60 h, the BM were separated into the OS and the VS and CD34^+^ recovery was calculated using FACS. Total eight mice were used. **g** SW480 colon cancer cells did not compete with CD34^+^ HSCs for BM niche. CD34^+^ HSCs were transferred with different numbers of SW480 cells into NOD/SCID mice. After engraftment for 60 h, CD34^+^ recovery was evaluated using FACS. Total nine mice were used

The BM is the primary niche for HSCs. Symptomatic MM is a gradual progression from MGUS or smoldering multiple myeloma (SMM), and the progression is pathologically determined based on the decreased proportion of normal plasma cells within the BM plasma cells [17]. To investigate MM competition with HSCs, we co-transferred CD34^+^ HSCs with increasing numbers of MM cells and calculated the engraftment capability of these cells within the same niche. During a short-term incubation, the recovery rates of CD34^+^ HSCs after 60 h were decreased in proportion to the increased numbers of MM cells injected in all three MM cell lines (Fig. 1a, Table S1, top), and their *R*^2^ values show an inverse correlation (Fig. 1a, Table S1, bottom). Since the injected cell numbers did not necessarily indicate that the cells home to the niche, we analyzed the *R*^2^ values based on the numbers of HSCs and MM cells engrafted in the BM, which showed a similar inverse correlation (Supplementary Figure 1a). A similar trend was observed during a long-term engraftment. The engraftment of HSCs after 16 weeks decreased in proportion to the increased numbers of MM cells injected (Fig. 1b, Table S2). The calculated *R*^2^ values based on the engrafted CD138^+^ MM cells yielded a similar inverse correlation (Supplementary Figure 1b).

Inhibition of HSC engraftment by MM cells further led to the suppression of HSC differentiation. The ability of HSCs to differentiation into leukocytes was greatly compromised when the MM cells were co-transplanted, and an increased inhibition was observed when higher numbers of MM cells were transferred (Fig. 1c, Table S2). We further investigated the dynamics between the HSCs and MM engraftment within the BM, and mainly focused on their occupancies in the OS or VS niches. In both OS and VS niches, a short-term (Fig. 1d, Table S3) and a long-term engraftment of HSCs (Fig. 1e, Table S4) was inversely affected by the increased MM cell numbers. The *R*^2^ values between the HSCs and MM showed an inverse correlation in both cases (Fig. 1d, e). Quantitative real-time PCR was performed to measure murine osteocalcin, BMP2, BMP4, and BMP7 levels in cells from OS and VS niches, which showed that these bone marker and morphogenic proteins were highly expressed in the cells from the OS niche (Supplementary Figure 1c). To validate the reduction of HSC recovery is specific to MM cells, we isolated normal CD138^+^ cells from healthy human BM and performed a competition assay between CD34^+^ HSC and normal CD138^+^ cells. FACS analysis demonstrated that increased numbers of normal plasma cells did not reduce CD34^+^ HSC engraftment (Fig. 1f). Also, we assessed whether other types of cancer cells could compete with HSC for the BM niche. The short-term competition assay showed that SW480, a colon cancer cell line, did not inhibit CD34^+^ HSC engraftment (Fig. 1g). Together, our data support that MM cells compete with HSCs for the BM niche support and affect the capacity of HSCs to differentiate into leukocytes.

### Quiescent MM cells prefer to reside in the OS niche

**Fig. 2:**
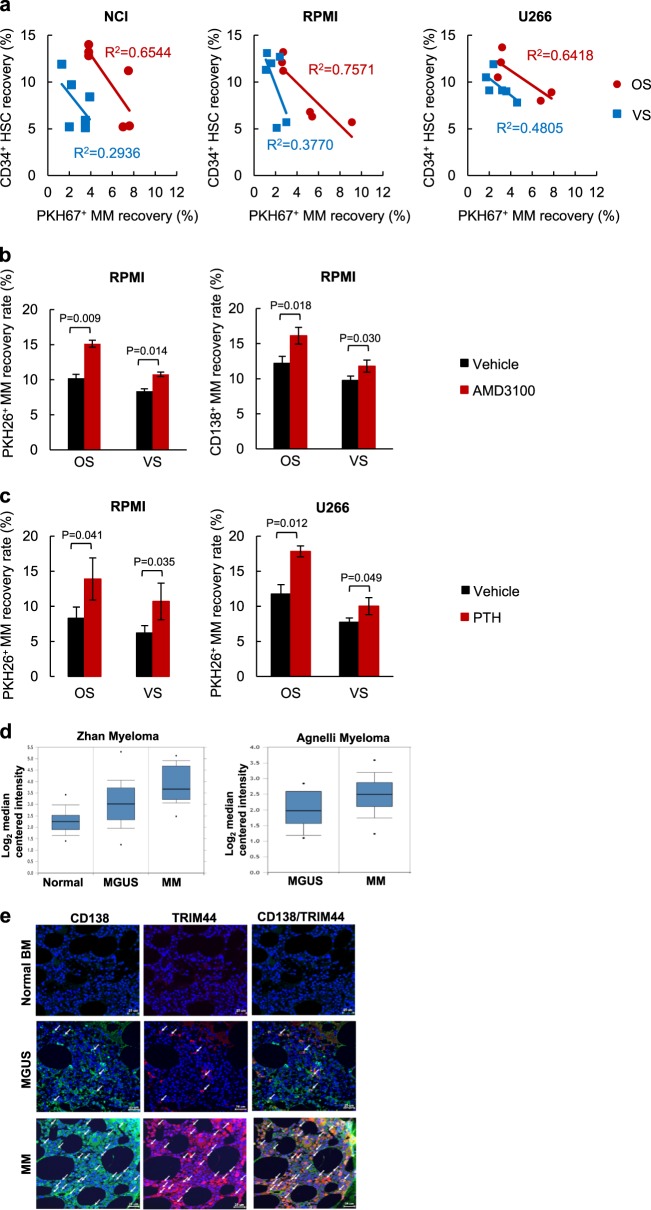

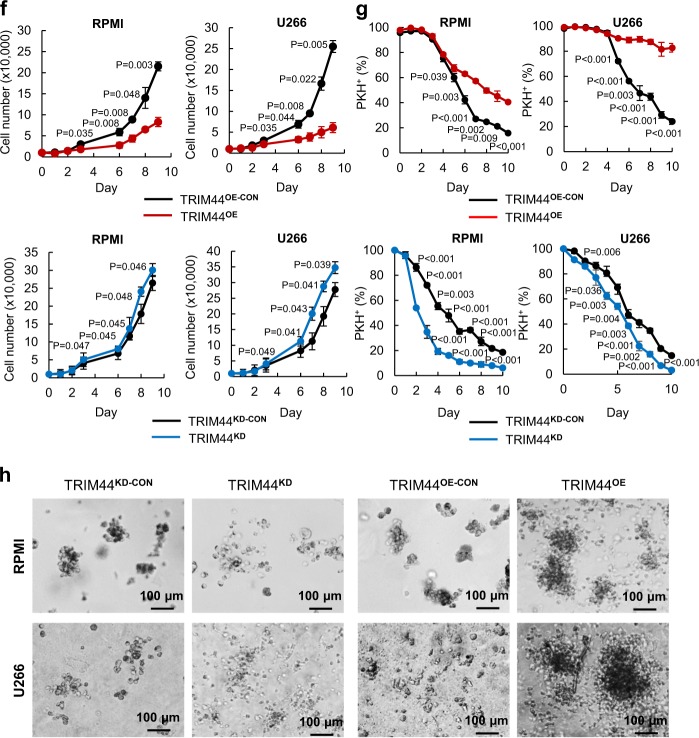
Quiescent MM cells prefer to reside within the OS niche. **a** The *R*^2^ values were calculated based on the number of CD34^+^ HSCs and the quiescent PKH67^+^ MM cells recovered in the OS or the VS niche. **b** The recovery rates of the PKH26^+^ MM cells in both the OS and VS niches were calculated in AMD3100 pre-treated mice. NOD/SCID mice were treated i.p. with AMD3100 for 5 days followed by an i.v. injection with PKH26 labeled RPMI cells (1 × 10^6^). After engraftment for 60 h, the MM cell recovery rates were calculated based on the PKH^+^ or CD138^+^ cells using FACS. Total six mice were used in this experiment. Detailed mice numbers in each experimental group are described in Table S5. **c** The number of PKH26^+^ MM cells recovered in the OS and VS niches was calculated in PTH-pre-treated mice. NOD/SCID mice were injected i.p. with PTH (50 mg/kg) or vehicle for 21 days. PKH26 labeled MM cells (RPMI and U266, 1 × 10^6^) were then i.v. transferred into mice and PKH^+^ cells were calculated 60 h post transplantation. The data are represented as the mean ± SD (error bars). The statistical significance was calculated using an unpaired Student’s *t*-test and is represented as a *P*-value. Total 12 mice were used in this experiment. Detailed mice numbers in each experimental group are described in Table S6. **d** TRIM44 expression increases in MM cells compared to normal BM or MGUS. Oncomine box plots of TRIM44 transcript levels based on two independent datasets from ref. 17, 18. For Zhan myeloma, 22 BM samples, 44 MGUS and 12 MM samples were analyzed and for Agnelli myeloma, 11 MGUS samples and 133 MM samples were used. The box plots display the normalized values, including the maximum value (the upper dot), the 90^th^ (the upper line outside the box), 75^th^ (the upper border of the box), median (the middle line within the box), 25^th^ (the bottom border of the box), and 10^th^ (the bottom line outside the box) percentiles and the minimum values (the bottom dot). The *P*-values were 1 × 10^-4^. **e** TRIM44 expression levels were compared in bone biopsies of normal human BM, MGUS, or MM patients. TRIM44 expression is increased in MM patients compared to normal BM or MGUS. TRIM44 (red), CD138 (green), and nucleus (blue) are shown. **f** TRIM44 expression in MM cells retards cell proliferation. TRIM44^OE-CON^ (control cells for TRIM44^OE^), TRIM44^OE^, TRIM44^KD-CON^ (control cells for TRIM44^KD^), and TRIM44^KD^ RPMI and U266 cells (10^4^) were plated at day 0. Cell counting was performed at the indicated times using trypan blue staining to exclude dead cells. The statistical significance was calculated by comparing cell proliferation at the same time point. **g** TRIM44^OE^ cells retained higher levels of PKH compared to TRIM44^OE-CON^ cells. TRIM44^OE-CON^ and TRIM44^OE^ RPMI cells were stained with PKH26, and >95% were PKH^+^ cells. PKH-labeled cells were then cultured, and the levels of dye retained in the cells were analyzed using FACS. The statistical significance was calculated by comparing PKH retentions between TRIM44^OE-CON^ and TRIM44^OE^ cells at the same time point. **h** TRIM44^OE^ cells increased colony formation in PHA-LCM methylcellulose medium. Different cell numbers (100, 50, 10, and 5 cells) were seeded and after 21 days, cell groupings of >40 cells were counted as a colony. The pictures were taken by an inverted microscope (×10). The data are represented as the mean ± SD (error bars). The statistical significance was calculated using an unpaired Student’s *t*-test and is represented as a *P*-value. Quantification of colony numbers were shown in Supplementary figure 2m

Since, we are primarily interested in quiescent MM cells and their ability to compete with HSCs, we labeled MM cells with the lipophilic dye, PKH as we previously described [2]. Proliferating cells lose PKH dye during division, while quiescent cells retain the dye [18]. The number of quiescent PKH^+^ cells that can be isolated from mice is low (5000–10,000/mouse), which makes it difficult to perform competitive assays with HSCs with varying numbers. Therefore, instead, we transferred HSCs (10^6^ cells) with PKH-labeled MM cells (10^6^ or 5 × 10^5^ cells) and allowed the MM cells to proliferate in vivo for three days, wherein they consequently lose PKH labeling upon proliferation. We then analyzed the engraftment of the quiescent cells at the OS or VS niches in the presence or absence of HSCs. Overall, there was a greater inverse correlation between the quiescent PKH^+^ MM cells and HSCs at the OS niche compared to those at the VS niche (Fig. 2a), confirming that the OS niche is the primary site for the quiescent MM cells. We evaluated levels of different B cell markers expressed in PKH^+^ and PKH^−^/CD138^+^ MM cells. Expression of CD19, CD20, and CD38, which are normally expressed in B cells, was increased in PKH^+^ MM cells from both OS and VS niches (Supplementary Figure 2a). We did not address the effects of quiescent MM cells on a long-term engraftment and the differentiation of CD34^+^ HSCs since it is difficult to recover reliable numbers of dye-retaining cells after 3–4 weeks due to the stability of PKH. We then pre-treated the mice with AMD3100, an antagonist of CXCR4, which mobilizes HSCs into the peripheral blood [19]. AMD3100 pretreatment increased both the PKH^+^ MM cell recovery rates in the OS niche as well as the VS niche (Fig. 2b, Table S5). We further manipulated the BM by pretreating the mice with parathyroid hormone (PTH) for 21 days to enhance targeting to the BM. The PKH^+^ cell recovery rate increased in both the OS and VS niches of PTH-pre-treated mice (Fig. 2c, Table S6).

### TRIM44 expression in MM cells slowed proliferation without cell death

Previously, we performed microarray analyses of PKH^+^ MM cells and PKH^−^CD138^+^ MM cells, which were isolated via FACS from ~40 NOD/SCID xenograft mice, to delineate the molecular mechanisms involved in quiescent MM cell targeting. The analyses of the genes that were differentially expressed in quiescent PKH^+^ cells, compared to proliferating cells (PKH^−^CD138^+^ cells) from all three niches revealed an upregulation of a relatively unknown gene named TRIM44. Quantitative real-time PCR (Supplementary Figure 2b) and immunoblots (Supplementary Figure 2c) further validated its upregulated expression in PKH^+^ cells from independent cell sources. A search of the integrated cancer microarray database (Oncomine) revealed that TRIM44 mRNA expression is significantly upregulated in MM compared to normal or MGUS (a precursor stage of MM) in two different datasets (Fig. 2d) [20, 21]. Immunofluorescence analysis of bone biopsies of MM, MGUS or healthy human BM showed that TRIM44 is highly expressed in MM compared to MGUS or healthy human BM (Fig. 2e). We further proceeded to characterize the potential roles of TRIM44 in MM development. TRIM44 protein levels are relatively low in different MM cells as well as in 293T cells (Supplementary Figures 3a and 3b). Since silencing TRIM44 using CRISPR-CAS9 methods (Supplementary Figure 3c) led to cell death (Supplementary Figure 3d), we overexpressed TRIM44 via a lentivirus-mediated overexpression (TRIM44^OE^) or silenced it via knockdown (TRIM44^KD^) to delineate the functions of TRIM44 (Supplementary Figures 3e-3g).

Control cells were infected with a proper control vector, which matched the infecting virus vector (TRIM44^OE-CON^ or TRIM44^KD-CON^). TRIM44 expression led to slowed cell proliferation, whereas TRIM44^KD^ MM cells did not influence MM cell proliferation (Fig. 2f), suggesting its functions in cell quiescence. A similar trend was observed in TRIM44^OE^ or TRIM44^KD^ 293T cells (Supplementary Figures 4a and 4b). To exclude the possibility that the retarded proliferation rates were not a result of higher apoptosis, cell apoptosis was determined. FACS analyses based on Annexin V and 7-AAD staining showed comparable apoptotic rates between TRIM44^OE^ and TRIM44^OE-CON^ cells (Supplementary Figure 4c). A comparable degree of cell apoptosis was also measured in TRIM44^OE^ and TRIM44^OE-CON^ 293T cells (Supplementary Figure 4d). To confirm this finding, we generated MM cells that expressed a tet-inducible TRIM44. TRIM44 expression was regulated by the addition or removal of doxycycline, and cell proliferation at the indicated durations was determined. Compared with the first 3 days without doxycycline treatment, at days 4–9 and 16–21, a tet-inducible TRIM44 slowed MM cell proliferation, and the removal of doxycycline at days 10–15 recovered MM cell proliferation (Supplementary Figure 5a). Doxycycline did not affect a proliferation rate in control MM cells (Supplementary Figure 5b), indicating changes of TRIM44 levels contributed MM quiescence. Since TRIM44^OE^ MM cells demonstrated cellular quiescence, we assessed whether TRIM44 expression led to increased PKH retention upon proliferation. Similar to what was observed in vivo, which became a basis of the TRIM44 discovery, TRIM44^OE^ MM cells retained higher amounts of PKH labeling compared to TRIM44^OE-CON^ cells (Fig. 2g). On the other hand, TRIM44 silencing reversed the trend (Fig. 2g). Cell cycle analyses using BrdU labeling revealed that S phase of TRIM44^OE^ cells was suppressed compared to TRIM44^KD^ cells (Supplementary Figure 5c). Moreover, the quiescent nature of TRIM44^OE^ MM cells resulted in increased colony formation in PHA-LCM methylcellulose-based medium (Fig. 2h, Supplementary Figure 6a), which was used to measure the clonogenicity of human B and T cells in MM [22].

### TRIM44 expression increases quiescent MM cell engraftment in the OS niche and increases the ability of MM to compete with HSCs

**Fig. 3:**
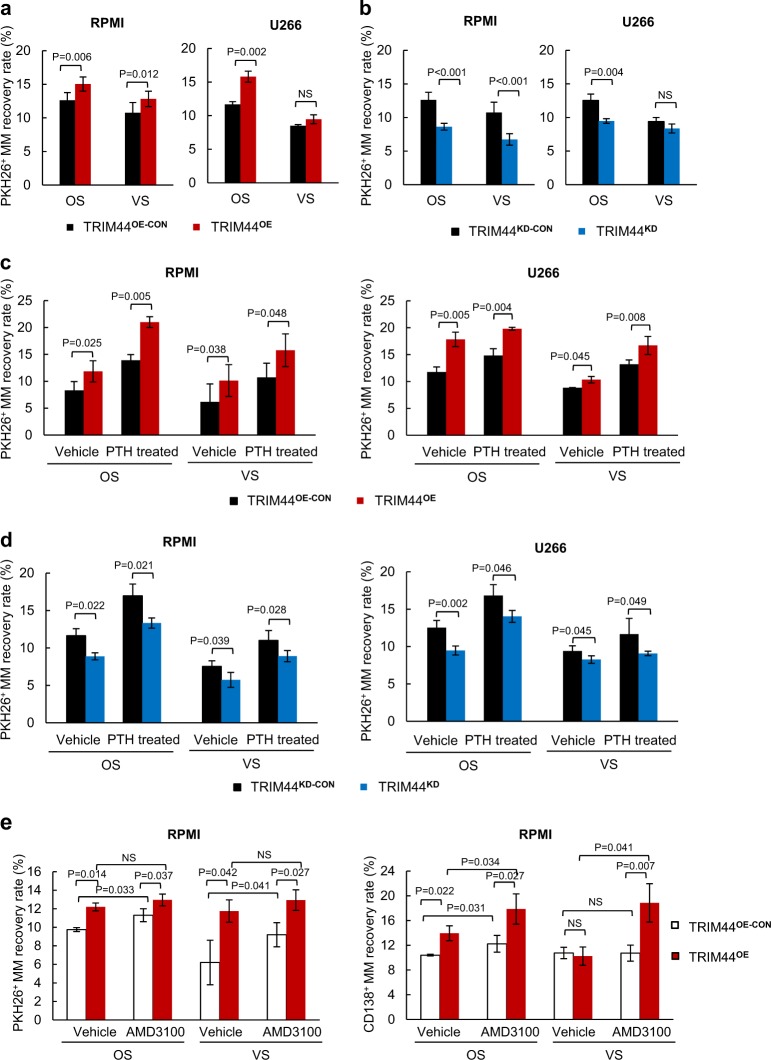
TRIM44 expression increases quiescent MM cell engraftment in the OS niche. **a** TRIM44^OE-CON^ and TRIM44^OE^ MM cells (RPMI, U266) were labeled with PKH26 and were i.v. injected into irradiated NOD/SCID mice. After 60 h, BM cells were separated into the OS or VS niches, and PKH^+^ MM cell numbers were calculated using FACS. Total 12 mice were used in this experiment. Detailed mice numbers in each experimental group are described in Table S7. **b** TRIM44^KD-CON^ and TRIM44^KD^ MM cells (RPMI, U266) were labeled with PKH26, and were i.v. injected into irradiated NOD/SCID mice. After 60 h, BM cells were separated into the OS or VS niches, and PKH^+^MM cell numbers were calculated using FACS. Total 12 mice were used in this experiment. Detailed mice numbers in each experimental group are described in Table S8. **c** NOD/SCID mice were i.p. injected with PTH (50 μg/kg) or vehicle for 21 days. PKH26 labeled TRIM44^OE–CON^ and TRIM44^OE^ MM cells (RPMI and U266) were then i.v. injected into the mice. After 60 h, BM cells were isolated as the OS and VS niche followed by FACS analyses. Total 24 mice were used in this experiment. Detailed mice numbers in each experimental group are described in Table S9. **d** NOD/SCID mice were i.p. injected with PTH or vehicle for 21 days. PKH26 labeled TRIM44^KD-CON^ and TRIM44^KD^ MM cells (RPMI and U266) were then i.v. injected into the mice. After 60 h, BM cells were isolated as the OS and VS nice followed by FACS analyses. Total 24 mice were used in this experiment. Detailed mice numbers in each experimental group are described in Table S9. **e** The recovery rates of PKH26^+^ TRIM44^OE-CON^ or TRIM44^OE^ MM cells in both the OS and VS niches were calculated in AMD3100 pre-treated mice. NOD/SCID mice were treated i.p. with AMD3100 for 5 days, followed by an i.v. injection with PKH26 labeled TRIM44^OE-CON^ or TRIM44^OE^ cells (1 × 10^6^). After engraftment for 60 h, MM cell recovery rates were calculated based on PKH^+^ staining using FACS. The statistical significance was calculated using an unpaired Student’s *t*-test and is represented as a *P*-value. Total 12 mice were used in this experiment. Detailed mice numbers in each experimental group are described in Table S10

We further analyzed the engraftment potential of the MM cells in different BM niches after TRIM44 expression. Compared to TRIM44^OE-CON^ cells, the recovery rate of quiescent PKH^+^TRIM44^OE^ MM cells increased especially in the OS niche (Fig. 3a, Table S7). On the other hand, knockdown of TRIM44 expression led to a decrease in the PKH^+^ MM engraftment in the OS niche (Fig. 3b, Table S8). Since the recovery rate of quiescent PKH^+^ cells increased with PTH injection (Fig. 2c), we tested whether TRIM44 expression further enhances its recovery numbers. Compared to the vehicle control, PTH treatment increased PKH26^+^ MM recovery, and TRIM44 expression dramatically increased the MM recovery rates in both the OS and VS niches (Fig. 3c, Table S9). On the other hand, knockdown of TRIM44 decreased the engraftment in the BM upon PTH treatment (Fig. 3d, Table S9). AMD3100 treatment also increased quiescent MM cell recovery in the OS and VS niches compared to the controls and vehicle treatment (Fig. 3e, Table S10).

**Fig. 4:**
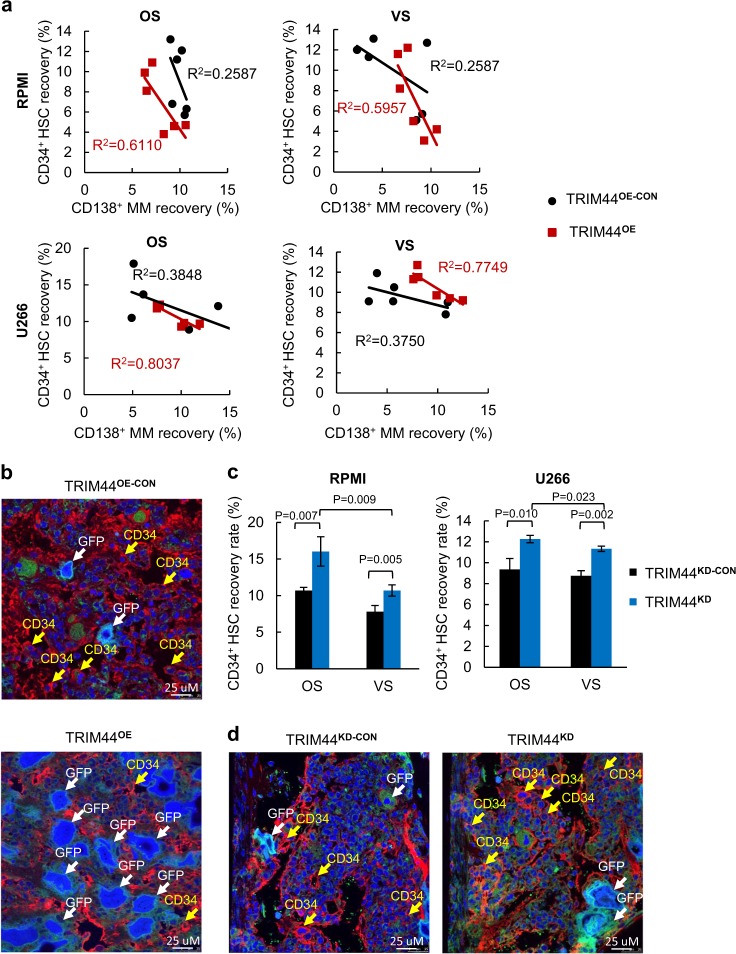
TRIM44 expression increases the ability of MM to compete with HSCs in the OS niche. **a** CD34^+^ HSCs were intravenously (i.v.) transferred into irradiated NOD/SCID mice with different numbers of TRIM44^OE-CON^ and TRIM44^OE^ MM cells (RPMI, U266, 0, 0.5 × 10^6^ or 1 × 10^6^ cells). After 60 h, the femurs were separated into the OS and VS niches. The calculated coefficient of determination (*R*^2^) showed an inverse correlation between the HSCs and MM cells in the OS niche. Total 24 mice were used in this experiment. Detailed mice numbers in each experimental group are described in Table S11. **b** Xenograft bones in **a** were examined using immunohistochemistry. Confocal microscopy imaging shows more CD34^+^ cells within the TRIM44^OE-CON^ compared to the TRIM44^OE^ injected mice. BM section stained with anti-CD34 and Draq5 (blue). The arrows indicate MM cells in GFP and HSCs in red in the BM. **c** CD34^+^ HSCs were intravenously (i.v.) transferred to irradiated NOD/SCID mice with different numbers of TRIM44^KD-CON^ and TRIM44^KD^ MM cells (RPMI, U266, 0, 0.5 × 10^6^ or 1 × 10^6^ cells). After 60 h, the femurs were separated into the OS and VS niches and the HSC recovery rates were calculated, which showed increased an HSC recovery when TRIM44 was silenced. Total 12 mice were used in this experiment. Detailed mice numbers in each experimental group are described in Table S12. **d** Confocal imaging of the immunohistochemistry of xenograft bones in **c**. More CD34^+^ cells were noticed in the femurs transferred with TRIM44^KD^ MM cells than with CD34^+^ HSCs

We then tested the ability of TRIM44^OE^ and TRIM44^KD^ MM cells to compete with HSCs. A greater inverse correlation (*R*^2^) was observed between CD34^+^ and TRIM44^OE^ MM cells compared to the values between CD34^+^ and TRIM44^OE-CON^ (*R*^2^ = 0.6110 vs. *R*^2 ^= 0.2587 in RPMI OS, *R*^2^ = 0.5957 vs. *R*^2^ = 0.2587 in RPMI VS, *R*^2^ = 0.8037 vs. *R*^2^ = 0.3848 in U266 OS, *R*^2^ = 0.7749 vs. *R*^2^ = 0.3750 in VS) (Fig. 4a, Table S11). In both the OS and VS niches, the engraftment increased upon TRIM44 expression (Fig. 4a). Immunohistochemistry results confirmed these data. There were more tumor cells (GFP^+^) present in the femurs of the mice transferred with TRIM44^OE^ MM cells compared to the controls (Fig. 4b). On the other hand, silencing TRIM44 increased CD34^+^HSC the recovery rates after a 3-day incubation (Fig. 4c, Table S12). Immunohistochemistry results also showed that more HSCs were present in the femurs of mice co-transplanted with TRIM44^KD^ MM cells (Fig. 4d).

**Fig. 5:**
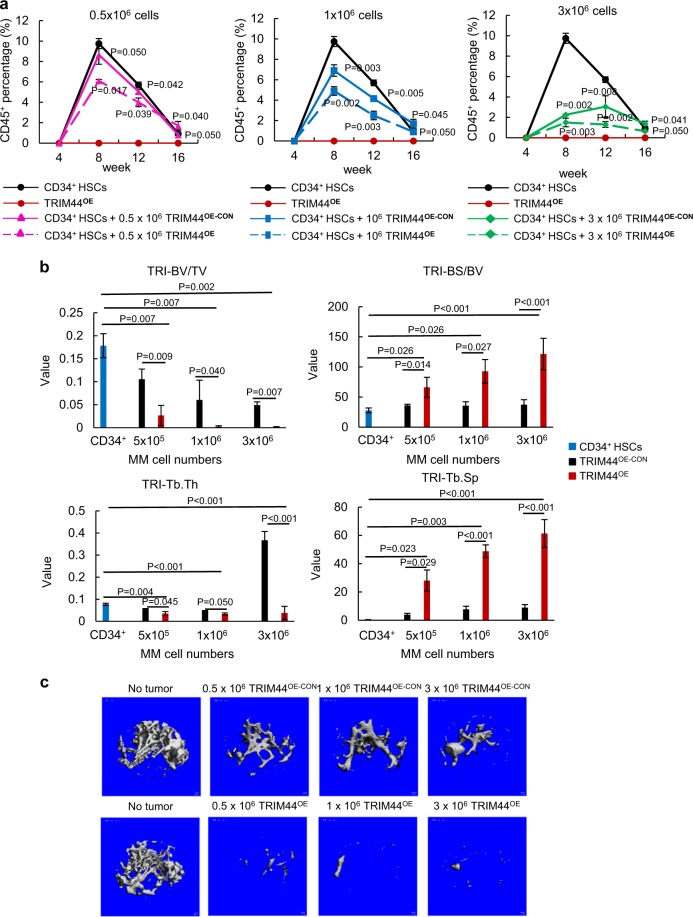
TRIM44 expression in MM inhibits HSC engraftment and enhances bone destruction. **a** TRIM44 expression in MM decreases HSC differentiation. CD34^+^ HSCs were i.v. transferred to NSG mice with different numbers of TRIM44^OE-CON^ and TRIM44^OE^ RPMI MM cells (0, 0.5 × 10^6^, 1 × 10^6^ or 3 × 10^6^ cells). At 8, 12, and 16 weeks, the CD45^+^ percentage in the peripheral blood of engrafted mice was determined using FACS. Total 21 mice were used in this experiment. Detailed mice numbers in each experimental group are described in Table S13. **b** Bone histomorphometry analyses of the tumor engrafted bones indicate accelerated bone destruction in TRIM44^OE^ mice. The engraftment method was described as **e**. At 16 weeks post-engraftment, the femur bone was isolated for histomorphometry analyses. TRI-BV/TV, bone volume; TRI-BS/BV, index of bone surface/bone volume ratio; TRI-Tb.Th, trabecular thickness; TRI-Tb.Sp, trabecular spacing; TRI-Tb.N, trabecular number. The data are represented as the mean ± SD (error bars). The statistical significance was calculated using an unpaired Student’s *t*-test and is represented as a *P*-value. Blue bars: CD34^+^, Black bars: TRIM44^OE-CON^, Red bars: TRIM44^OE^. **c** 2D analyses of the bones (femurs) showing increased bone destruction in TRIM44^OE^ cell injected mice after 16 weeks

We further analyzed the effects of TRIM44 expression in MM cells during a long-term engraftment with HSCs. A differential number of TRIM44^OE-CON^ or TRIM44^OE^ MM cells was co-transferred with CD34^+^ HSCs into NSG mice. At 8, 12, and 16 weeks post transplantation, the numbers of human CD45^+^ cells in the peripheral blood were determined. Transferring TRIM44^OE^ MM cells with HSCs further reduced the number of CD45^+^ cells in the blood compared to TRIM44^OE-CON^ (Fig. 5a, Table S13), suggesting that TRIM44^OE^ MM cells effectively suppressed HSC function. At 16 weeks post transplantation, the total CD45^+^ cells recovered in the OS niche were reduced upon MM co-transplantation and was further reduced upon TRIM44 expression (Fig. 5a). Collectively, the in vivo engraftment competitive assays demonstrated that TRIM44^OE^ enhances the engraftment of quiescent MM cells in the OS niche, which further interfere HSC long-term engraftment and differentiation.

Bone lytic legions are the main symptoms of MM patients. Therefore, we conducted bone histomorphometry analyses to examine the effects of TRIM44 expression on bone destruction. The indexes of bone structure, including bone volume (TRI-BV/TV) and trabecular thickness (TRI-Tb.Th), were significantly decreased, while the ratio of the bone surface/bone volume (TRI-BS/BV) and trabecular spacing (TRI-Tb.Sp) were increased, in TRIM44^OE^ MM-engrafted mice (Fig. 5b). Representative 3D structure analyses showed that over-expressing TRIM44 in MM increased bone destruction of mice compared to the controls (Fig. 5c). TRIM44 silencing in TRIM44^OE^ MM cells delayed bone destruction in xenograft mice (Supplementary Figure 6b). Since TRIM44^OE^ MM cells increased engraftment to the BM with increased bone destruction, we analyzed the interaction between TRIM44^OE^ MM cells and human BM stromal cells (HS5 or HS27). The adherent cell numbers were determined based on fluorescence intensity. MM cell adhesion to HS5 or HS27 increased upon TRIM44 expression (Supplementary Figure 7a), whereas adhesion was decreased upon TRIM44 silencing (Supplementary Figure 7b). Similarly, PKH^+^ cells isolated from the OS and VS niches of xenografted mice showed an increased adhesion capability compared to PKH^-^CD138^+^ cells (Supplementary Figure 7c). Together, our data support that TRIM44 expression increases MM targeting to the BM, and its increased interaction with BM stromal cells could lead to increased bone destruction in vivo.

### TRIM44 stabilizes HIF-1α via deubiquitination

Unlike other TRIM family member proteins, TRIM44 contains a ZF-UBP domain in the N-terminal region instead of a RING domain. UBP domains are often found in deubiquitinating enzymes, and the ZF-UBP domain in TRIM44 is reported to function as a deubiquitinating enzyme [23, 24]. Stabilization of HIF-1α protein increases HSC quiescence in vivo, whereas HIF-1α deficiency reduces HSC quiescence. This suggests the precise regulation of HIF-1α level is essential for maintenance of HSC quiescence in BM [25, 26]. Similar with HSC, hypoxia induces immature and stem-like phenotypes in MM cells by decreasing MM cell proliferation and acquiring a quiescent state without apoptosis [27, 28]. Since TRIM44 is overexpressed in quiescent MM cells in the hypoxic BM niche, we investigated whether TRIM44 acts as a deubiquitinase for HIF-1α to bolster its stabilization.

**Fig. 6:**
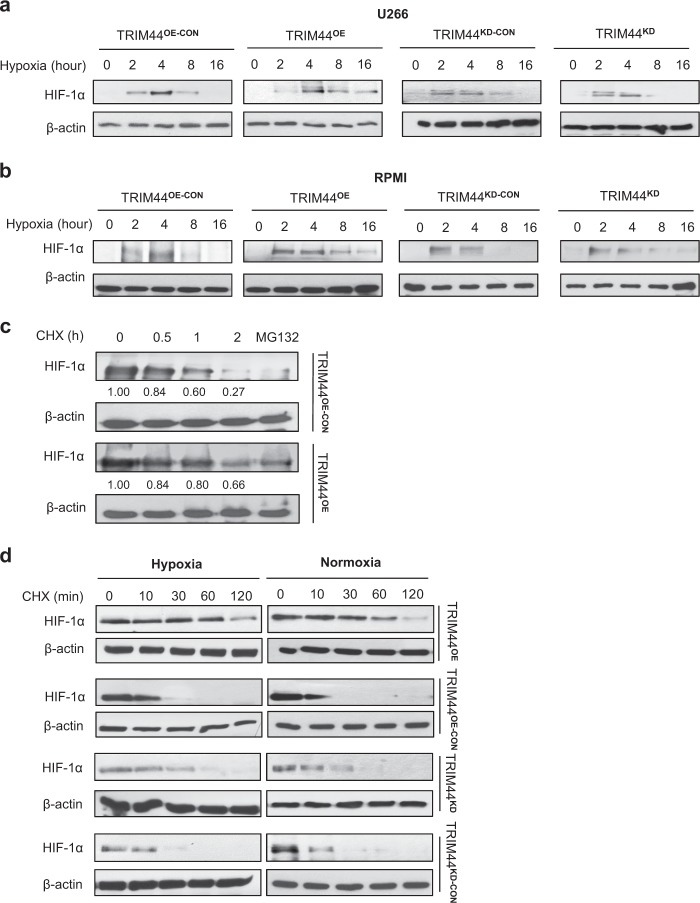
TRIM44 expression enhances HIF-1α stability and increases its downstream gene expression. **a** and **b** TRIM44^OE-CON^, TRIM44^OE^, TRIM44^KD-CON^ and TRIM44^KD^ U266 cells (**a**) or RPMI (**b**) were cultured in hypoxia conditions (1% O_2_) for the indicated hours. HIF-1α levels were determined using immunoblotting, which was normalized using β-actin. **c** TRIM44^OE-CON^ and TRIM44^OE^ 293T cells were transfected with HA-HIF-1α. After 48 h, the cells were treated with 50 μg/ml cycloheximide (CHX) for the indicated hours. HIF-1α levels were determined by immunoblotting and were normalized by β-actin. **d** TRIM44^OE-CON^, TRIM44^OE^, TRIM44^KD-CON^, and TRIM44^KD^ RPMI MM cells were incubated in hypoxia conditions for 4 h. Subsequently, cycloheximide (CHX, 50 μg/ml) was added for 0, 10, 30, 60, and 120 min under normoxia or hypoxia conditions. Cell lysates were then subjected to immunoblotting analyses using HIF-1α. Quantitation of HIF-1α expression was normalized by β-actin and shown in Supplementary Figure S4d

To evaluate whether HIF-1α is the target of TRIM44 deubiquitinating activity, we first measured HIF-1α levels in TRIM44^OE^ and TRIM44^KD^ MM cells, and in their respective control cells. Compared with TRIM44^OE-CON^ MM cells, TRIM44^OE^ cells increased HIF-1α expression under hypoxia (Fig. 6a, b). On the other hand, silencing TRIM44 decreased the induction of HIF-1α (Fig. 6a, b). When hypoxia was induced in 293T cells using a cobalt (II) chloride treatment (200 μM, CoCl_2_), HIF-1α was increased in TRIM44^OE^ cells (Supplementary Figure 8a). The expression of well-known HIF-1α downstream genes, such as vascular endothelial growth factor (VEGF), glucose transporter 1 (GLUT1), and matrix metallopeptidase 9 (MMP-9) increased in TRIM44^OE^ MM cells, while their expression levels were decreased in TRIM44^KD^ cells (Supplementary Figure 8b). The protein levels of VEGF, GLUT1, and MMP-9 showed similar trends in both TRIM44^OE^ and TRIM44^KD^ MM cells (Supplementary Figure 8c).

Since deubiquitination prevents proteins from undergoing proteasome-mediated degradation, and HIF-1α is a potential target modified by TRIM44, we analyzed the half-life of the HIF-1α protein. Compared with TRIM44^OE-CON^ cells, TRIM44^OE^ cells displayed a HIF-1α protein with a longer half-life (Fig. 6c). We then induced HIF-1α in TRIM44 manipulated MM cells by incubating cells for 4 h in hypoxia followed by incubating cells either hypoxia or normoxia with a protein synthesis inhibitor, cycloheximide. Compared to TRIM44^OE-CON^, TRIM44^OE^ cells showed a longer HIF-1α half-life (Fig. 6d, Supplementary Figure 9a). To investigate roles of HIF-1α in MM, we treated TRIM44^OE^ MM cells with KC7F2, a HIF-1α inhibitor. KC7F2 treatment reduced TRIM44^OE^ viability up to 60% over 8 days, suggesting a critical role of HIF-1α in MM survival (Supplementary Figure 9b).

### TRIM44 deubiquitinates HIF-1α via K48

To determine whether TRIM44 reduced HIF-1α polyubiquitination, 293T cells were transfected with HIF-1α and ubiquitin and were treated with MG132 to accumulated polyubiquitinated proteins. TRIM44 transfection reduced polyubiquitinated HIF-1α (lane 3), compared to no TRIM44 transfection (lane 1 and 2), and simultaneously increased wild-type HIF-1α (lane 3 and 4, arrow) (Supplementary Figure 9c). MG132 treatment did not cause the accumulation of polyubiquitinated HIF-1α with TRIM44 (lane 4), suggesting that TRIM44 decreases HIF-1α polyubiquitination (Supplementary Figure 9c). To further validate the deubiquitinase function of TRIM44 in polyubiquitinated HIF-1α, von Hippel-Lindau tumor suppressor (VHL), which is an E3 ligase for HIF-1α, was co-transfected into the 293T cells in the presence or absence of TRIM44. TRIM44 decreased VHL-mediated HIF-1α polyubiquitination, supporting that TRIM44 reduces HIF-1α polyubiquitination (Supplementary Figure 9d). Proteins targeted for proteasomal degradation are associated with lysine-48 (K48) linked polyubiquitination, and a lysine-63 (K63) linked ubiquitin chain regulates protein function through conformational changes, protein–protein interactions, or changes in subcellular location [29]. To clarify whether TRIM44 reduced proteasome-associated polyubiquitination, K48-ubiquitin or K63-ubiquitin was co-transfected with HIF-1α and TRIM44 into 293T cells. K48-linked polyubiquitination of HIF-1α was reduced by TRIM44, whereas K63-linked polyubiquitination of HIF-1α did not change (Supplementary Figure 10a). A similar result was observed when TRIM44^OE^ MM cells were used (Supplementary Figure 10b). Decreased K48-linked polyubiquitination of HIF-1α was observed in TRIM44^OE^ cells, supporting that TRIM44 decreases K48-linked polyubiquitination of HIF-1α.

### TRIM44 interacts with HIF-1α for direct deubiquitination

**Fig. 7:**
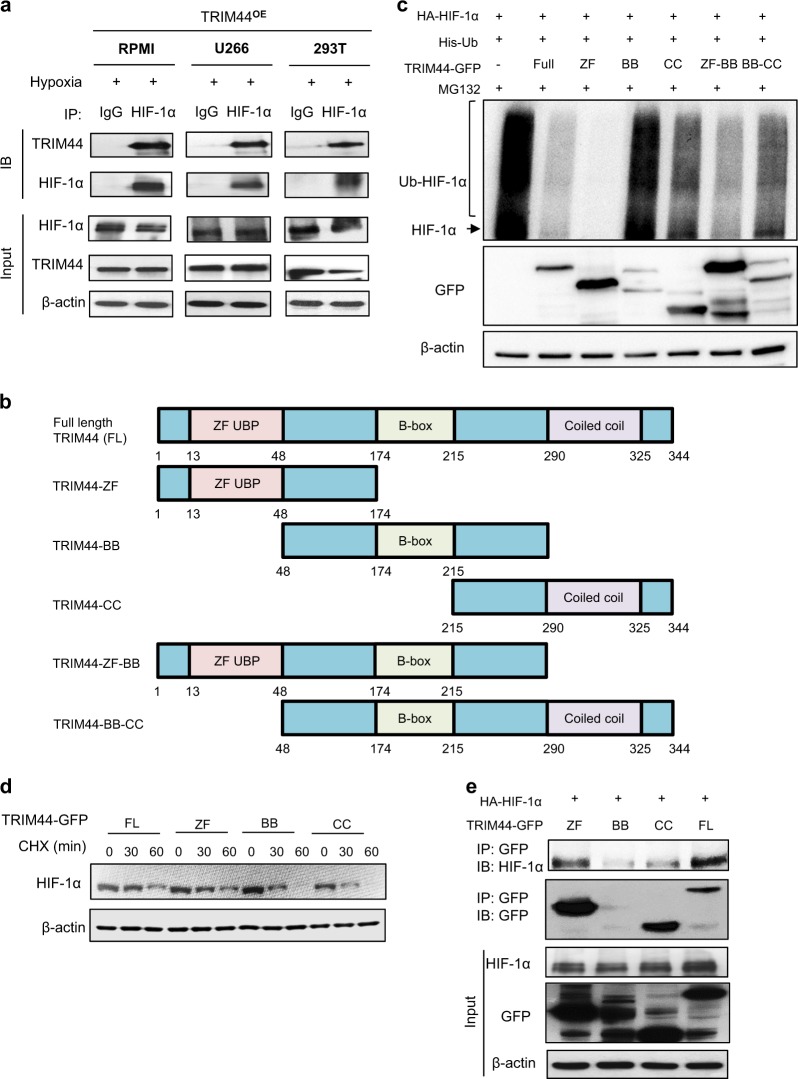
TRIM44 deubiquitinates HIF-1α via zinc finger domains. **a** TRIM44^OE^ RPMI, U266, and 293T cells were cultured in hypoxia for 4 h. The cells were immunoprecipitated using IgG or HIF-1α antibodies and were then immunoblotted using TRIM44 or HIF-1α antibodies. **b** Diagram of functional domains contained in truncated TRIM44. **c** Identification of the TRIM44 functional domain for HIF-1α deubiquitination. A combination of HA-HIF-1α, His-Ub, and various truncated forms of TRIM44-GFP (Full, ZF:zinc finger only, BB: B box domain only, CC: coiled coil domain only, ZF-BB: zinc finger and BB domains, BB-CC: BB domain and CC domain) were expressed in 293T cells. The cells were treated with MG132 for 6 h before collection. Ubiquitinated HIF-1α (over 120 kD) was measured by immunoblotting. **d** 293T cells were transfected with HA-HIF-1α combined with full length TRIM44 (FL), or the ZF, BB, or CC domain of TRIM44. After 48 h, the cells were treated for 0, 30, or 60 min with cycloheximide (CHX). HIF-1α levels were determined by immunoblotting and were normalized by β-actin. **e** 293T cells were transfected with HA-HIF-1α in the presence of the ZF-UBP domain (ZF), B-box domain (BB), or coiled-coil domain (CC) of TRIM44. Cell lysates were immunoprecipitated with anti-GFP. Immunoblotting was performed to determine the interaction between HIF-1α and the TRIM44 domains

To determine whether TRIM44 directly interacts with HIF-1α for deubiquitination, immunoprecipitation analyses were performed using TRIM44^OE^ MM cells cultured in hypoxia. HIF-1α was pulled down together with TRIM44 in TRIM44^OE^ MM cells as well as in 293T cells under hypoxia (Fig. 7a). Exogenous TRIM44 expression in 293T cells also interacted with HIF-1α in the same cells (Supplementary Figure 10c). In 293T cells cultured in a hypoxia condition, endogenous HIF-1α was immunoprecipitated together with endogenous TRIM44 (Supplementary Figure 10d), which confirmed a direct interaction between HIF-1α and TRIM44.

To identify the functional domains for HIF-1α deubiquitination in TRIM44, we generated truncated TRIM44 constructs that lacked the ZF domain, BB domain, CC domain, ZF-BB tandem domain, or BB-CC tandem domain (Fig. 7b). Only the truncated TRIM44 domains containing ZF domains inhibited HIF-1α polyubiquitination (Fig. 7c). Compared with the BB, CC, or BB-CC domain alone, the ZF-BB tandem domain could reduce HIF-1α polyubiquitination, indicating that the ZF domain is critical for HIF-1α deubiquitination (Fig. 7c). We also investigated whether the ZF domain extends the half-life of the HIF-1α protein. Compared with the BB domain and CC domain, full-length TRIM44 and the ZF domain significantly extended the half-life of HIF-1α after 30 min of cycloheximide treatment (Fig. 7d). To investigate which functional domains of TRIM44 interact with HIF-1α, we co-transfected HIF-1α with the ZF, BB, or CC domain of TRIM44 into 293T cells. The immunoprecipitation showed that HIF-1α interacts with the BB domain of TRIM44 (Fig. 7e). Taken together, our data support that TRIM44 interacts with HIF-1α, preventing HIF-1α degradation by deubiquitination.

## Discussion

Even though many studies have aimed to understand the MM cell interaction with the BM microenvironment, our previous report identifying the OS niche as a niche for quiescent MM cells was one of the first attempts investigating two distinct niches that regulate MM cell activity. Since MM cells mainly progress within the BM, they are a good model for understanding cancer spreads to the BM. Understanding how quiescent tumor cells mobilize from the BM niche to circulation, and investigating their roles in tumor relapse after chemotherapies will have an important clinical relevance. Depending on the cancer type, disseminated cancer cells occupy a different BM niche. For example, prostate cancer cells disseminate and occupy the OS niche, which facilitates its metastasis [30]. On the other hand, disseminated breast cancer cells prefer the perivascular niche of the BM [31]. Similar to HSCs, the local microenvironment the MM cell reside affected their characteristics, such as tumorigenic activities and stem-like features [2]. Quiescent MM cells isolated from the OS niche form colonies in the methylcellulose medium, whereas quiescent cells from the VS niche did not form colonies [2].

Similar with our findings, disseminated prostate cancer cells inhibited CD34^+^ HSC engraftment and competed for HSC niche, whereas non-metastatic transformed prostate epithelial cells did not repress CD34^+^ HSC engraftment [30]. Low-HSC frequency in acute myeloid leukemia correlated with poor overall and disease-free survivals of patients [32]. This data suggest that competition between leukemic stem cells and HSCs for niche spaces is the main factor for declining HSC numbers before relapse. Given that most MM patients will undergo an autologous stem cell transplantation after standard therapies and eventually most patients relapse, understanding how quiescent cells hide in the OS niche to avoid therapies will have significant therapeutic implications.

The tripartite motif (TRIM) protein family is characterized by an N-terminal RING finger domain, B-box, and coiled-coil domains and is involved in a broad range of biological process [3, 33], and their altered expression and function are associated with pathologies, such as neurodegenerative diseases [34, 35], viral infection, and cancer. [36, 37] TRIM family genes are involved in cancer as either oncogenes or tumor-suppressors. For example, in gastric cancer overexpression of TRIM28 and TRIM29 is associated with increased cell proliferation and a poor prognosis in patients [38, 39]. TRIM13, TRIM19, TRIM24, and TRIM28 regulate the expression levels of p53 by controlling p53 ubiquitination and degradation via MDM2 or by inducing stabilization of p53 by ubiquitination of MDM2 [40].

Even though the TRIM family contains more than ~80 proteins in humans, TRIM44 is the only deubiquitinase in the family. Unlike other TRIM family member proteins, TRIM44 contains a zinc finger UBP in the N-terminal instead of a RING domain, and UBP domains are often found in deubiquitinating enzymes [7]. Since it was first identified in the mouse brain, only a few studies have analyzed the functions of TRIM44, and these have mostly been in cancers or virus infection (17 total publications as of 2017). Overexpression of TRIM44 is reported in 16% of epithelial cancers including gastric cancers [12] and breast cancers [13]. In non-small cell lung cancer, upregulated TRIM44 promotes the migration and invasion of cancer cells via NF-kB signaling [9, 41]. However, the precise functions of TRIM44 in cancer remain unclear. Quiescent PKH^+^ MM cells isolated from the OS niche contained elevated levels of TRIM44, indicating that TRIM44 transforms MM cells into a quiescent phenotype and subsequently enhances MM cell occupancy in BM niche. Our data support the oncogenic role of TRIM44 in MM and their roles in the maintenance of MM dormancy in BM niches.

In HSCs, HIF-1α is essential for the maintenance of HSC quiescence [25, 26]. HIF-1α deficiency leads to a loss of cell cycle quiescence in HSCs and deceasing HSCs numbers upon BM stress or aging, whereas overstabilization of HIF-1α by a deficiency of VHL induces the quiescence in HSCs. HIF-1α also critically supports survival and self-renewal in cancer stem cells and disseminated tumor cells in metastasis sites. Hypoxia microenvironment upregulates quiescence and dormancy gene expression in tumor cells and induces quiescence of disseminated prostate cells in metastatic sites [42]. These disseminated tumor cells with low-proliferative property exhibit resistance to chemotherapy. Since, the hypoxic microenvironment in BM niche is important for maintenance and control of quiescent phenotype in both HSC and cancer cells, MM cells could take advantage of the hypoxic BM microenvironment for their tumor initiation and survival. MM initiation occurs in the hypoxic BM niche, followed by stimulated angiogenesis to increase oxygen tension to support continuing MM cell growth [43]. In HSCs, the upregulated oxygen level in BM microenvironment drives HSC differentiation and dislodgement of HSC from the BM niche.

We discovered novel roles of TRIM44 in maintaining MM quiescence. Our data support that MM cells increased BM occupancy upon TRIM44 expression, which reversed upon TRIM44 knockdown. Increased BM occupancy further enhanced MM occupancy within the BM and increased competition with HSCs. Increased BM occupancy in TRIM44^OE^ MM cells affected HSC functions including their differentiation.

We also discovered that TRIM44 significantly enhances viability and quiescence of MM cells under hypoxia. TRIM44 stabilizes HIF-1α via deubiquitination, which is a key transcription factor that regulates MM tumor growth, angiogenesis, and bone destruction [44, 45]. In our study, reduced HIF-1α polyubiquitination by TRIM44 overexpression was associated with HIF-1α protein levels and an extended half-life in MM cells. Furthermore, HIF-1α stabilized by TRIM44 was supported by the upregulation of HIF-1α target genes, including VEGF and GLUT1, whereas knockdown of TRIM44 obviously downregulated these gene expression levels. Our data support that TRIM44 prevents the proteasomal degradation of HIF-1α and promotes the quiescent phenotype of MM cells.

Dysregulation of protein ubiquitination and deubiquitination pathways is one of the pathological features of MM [46]. MM cells, which is a malignant form of antibody-secreting plasma cells, constitutively suffer increased ER stress and hence are sensitive to compounds targeting protein homeostasis, such as proteasome inhibitors. Bortezomib is one example of how targeting the ubiquitin-proteasome pathway suppresses MM growth [47]. In MM cells, proteasome-associated deubiquitinating enzymes play a central role in modulating the unfolded protein response. Recent studies show that inhibitors targeting enzymes that modulate protein ubiquitination/deubiquitination upstream of the proteasome inhibit MM tumor growth and overcome bortezomib resistance [48]. Since the upregulation of the deubiquitinase TRIM44 in quiescent MM cells supports their growth in the BM niche and drug resistance, the development of an inhibitor targeting TRIM44 could benefit to defeat drug resistance and provide the framework for a synergistic therapy to improve the outcome of MM patients.

In summary, our findings support that TRIM44 is an important factor that regulates the quiescence property of MM cells. Our report is the first to show that TRIM44 stabilizes HIF-1α to maintain its stability and in turn supports the survival of quiescent MM. Understanding the mechanism of TRIM44 regulation could promote the development of specific deubiquitinase inhibitors in MM therapy in the future.

## Materials and methods

### Isolation of human CD34^+^ hematopoietic stem cells (HSCs)

CD34^+^ HSCs were isolated from human cord blood obtained from Department of Stem Cell Transplantation Research Lab, MD Anderson Cancer Center. Cell suspension from cord blood was incubated with human CD34 MicroBeads (Miltenyi Biotec). CD34^+^ HSCs were enriched via positive selection using Miltenyi Biotec MiniMACS Separator Columns (Miltenyi Biotec) according to the manufacturer protocol.

### PKH staining

MM cells were stained with PKH (Sigma-Aldrich) for 5 min according to manufacturer protocol. To ensure ≥99% cells were PKH^+^, the PKH-stained cells were examined for PKH^+^ percentage by using FACS analysis. For xenograftment, the 10^6^ PKH-stained MM cells were i.v. injected into irradiated (225 cGy) NOD/SCID mice, and the mice were killed after 60 h. For PKH retention assay, percentages of PKH-prestained MM cells were analyzed using FACS analysis at the indicated time.

### In vivo competitive engraftment assay

CD34^+^ HSCs were isolated from human cord blood and enriched via positive selection. NCI-H929, RPMI8226, and U266 cells were stained with PKH for 5 min according to manufacturer protocol. Stained cells were examined for PKH intensity using FACS analysis to ensure that >99% cells were PKH positive. Stained MM cells and HSCs were mixed and injected into sublethally irradiated NOD/SCID mice via i.v. injection. The mice were killed after 60 h for examination of BM.

### Ubiquitination assay and half-life assay

HEK293T cells were plated in six-well plate before the day of transfection. HA-HIF-1α, His-Ub, and TRIM44-GFP plasmids were carried by calcium phosphate for transient expression. At 48 h post transfection, cells were treated with 5 μM MG132 for 6 h. Cell lysates were collected for immunoblotting analysis. For half-life assay, HEK293T cells were transfected with HA-HIF-1α. After 48 h, cells were treated 50 μg/mL cycloheximide for indicated time, and cell lysates were collected for immunoblotting analysis.

## Electronic supplementary material

Supplemental_Figures

Supplemental Table

Supplemental Methods
