# Supplementary material for: TRIM44 promotes quiescent multiple myeloma cell occupancy and survival in the osteoblastic niche via HIF-1α stabilization
Source: Leukemia. 2018 Aug 8;33(2):469–86. doi: 10.1038/s41375-018-0222-x (PMC6365383; doi:10.1038/s41375-018-0222-x)
Supplement: Supplementary file 1 — Supplemental_Figures [file 41375_2018_222_MOESM1_ESM.pdf]

Supplementary Figure 1

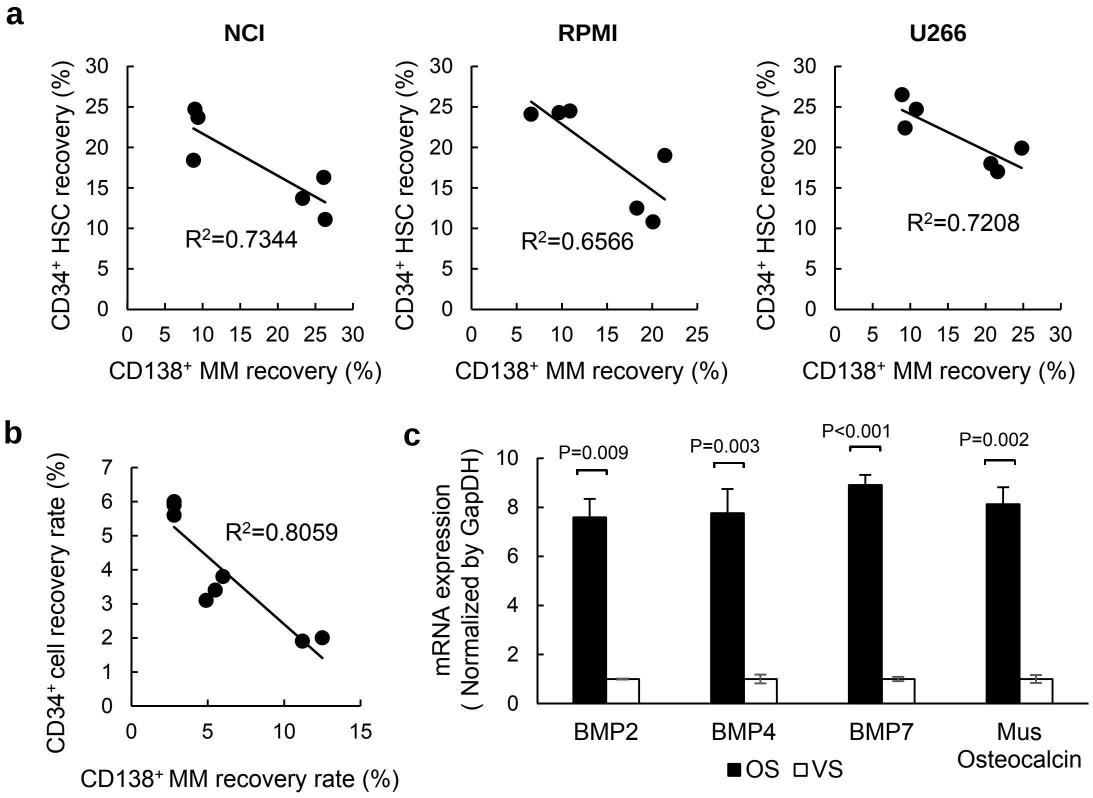

Figure S1. TRIM44 expression increases in quiescent MM cells isolated from the OS niche of BM. (a) $CD34^+$ HSCs and MM cells were intravenously (i.v.) transferred into irradiated NOD/SCID mice with different numbers of MM cells (NCI, RPMI, U266, 0, 0.5 x 10^6 or 1 x 10^6 cells). After 60 hours, the femurs were isolated and $CD34^+$ cell recovery and $CD138^+$ cell recovery (engrafted cell numbers) were calculated using FACS analyses. The coefficient of determination (R^2) showed an inverse correlation between HSC and MM. (b) The R^2 values were calculated between engrafted $CD34^+$ cells and engrafted $CD138^+$ MM cells, which showed an inverse correlation. (c) Expression levels of osteocalcin, BMP2, BMP4, and BMP7 in cells from the OS niche and the VS niche. Primers for mouse osteocalcin: F-CCCTGCTTGTGACGAGCTAT; R-ACTTGCAGGGCAGAGAGAGA. BMP2: F-TGGAAGTGGCCCATTTAGAG; R-TGACGCTTTTCTCGTTTGTG. BMP4: F-TGATACCTGAGACCGGGAAG; R-AGCCGGTAAAGATCCCTCAT. BMP7: F-GAAAACAGCAGCAGTGACCA; R-GGTGGCGTTCATGTAGGAGT.

Supplementary Figure 2

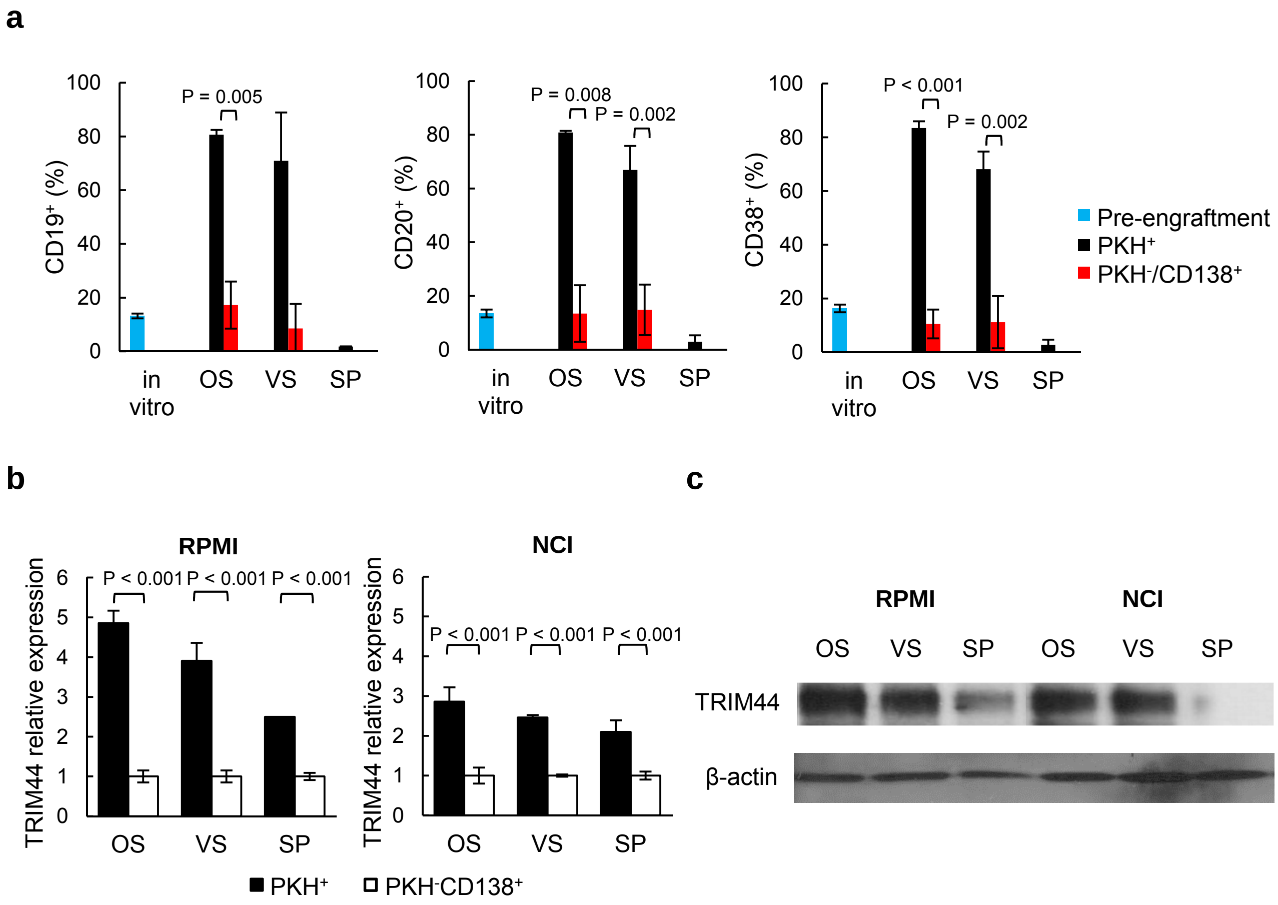

Figure S2. (a) Expressions of CD19, CD20, CD38 in pre-engrafted, post-engrafted PKH^{+} and PKH^{-}/CD138^{+} MM cells. RPMI cells were pre-labeled with PKH and engrafted via i.v. injection (n = 3). Mouse bone marrow cells were isolated after 60 h and analyzed CD19, CD20, and CD38 in PKH^{+} and PKH^{-}/CD138^{+} populations using FACS. (b) TRIM44 expression was upregulated in PKH^{+} MM cells. PKH67-labelled RPMI and NCI cells were transferred into irradiated NOD/SCID mice. After 60 hours, PKH^{+} and PKH^{-}CD138^{+} cells were isolated from the OS, VS of the BM and spleens. Quantitative RT-PCR was performed to determine the level of TRIM44. (c) Increased TRIM44 expression in the OS niche of the BM. TRIM44 protein expression was evaluated in the OS, VS, and spleens using immunoblots.

Supplementary Figure 3

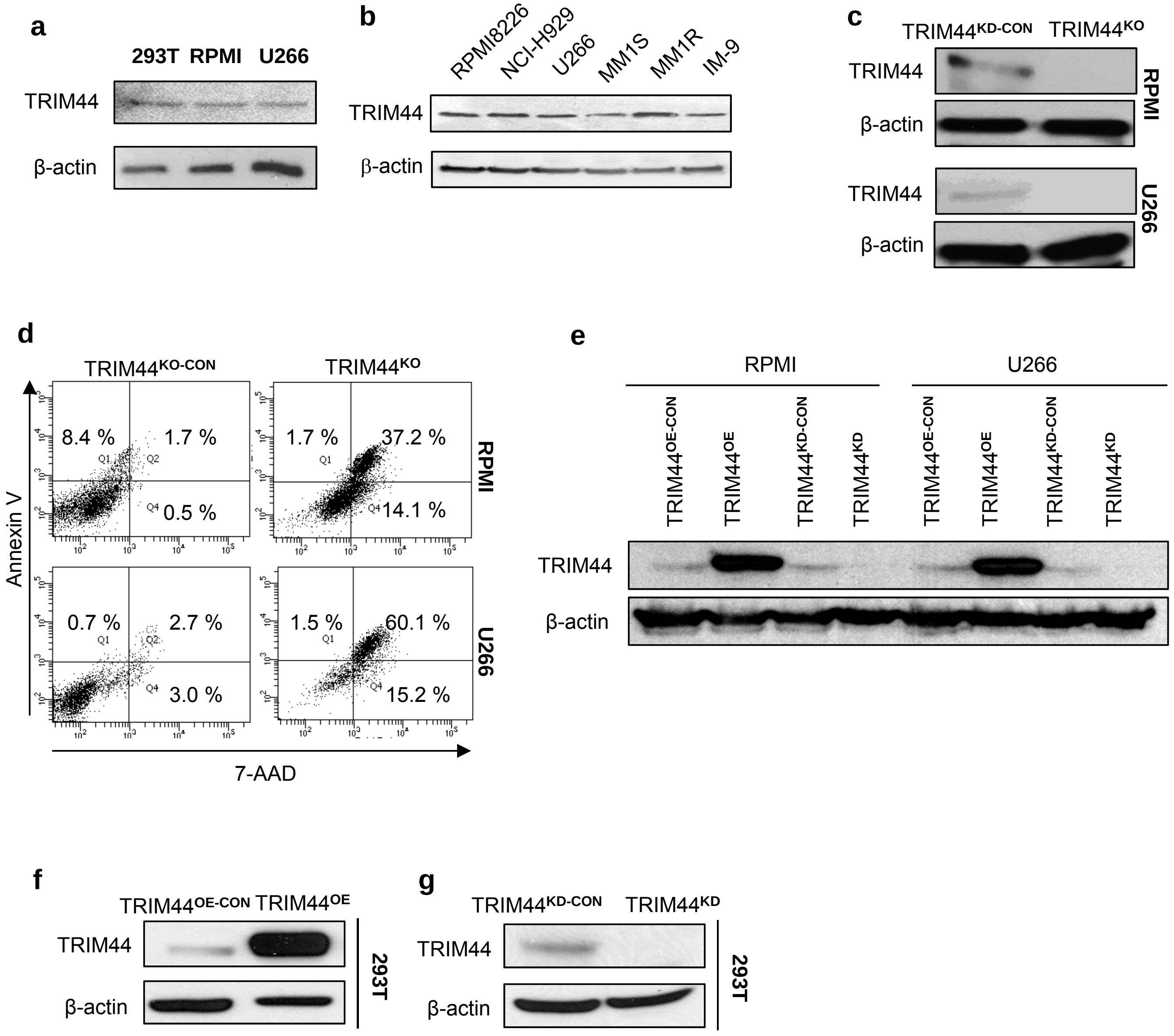

Figure S3. TRIM44 silencing in MM cells leads to cell death. (a) Endogenous TRIM44 protein levels in RPMI, U266, and HEK293 cells. (b) Endogenous TRIM44 protein levels in RPMI, NCI, U266, MM1S, MM1R and IM9 were examined using immunoblots. (c) Silencing TRIM44 (TRIM44^{KO} using a CRISPR-CAS9) in MM cells leads to cell death. (d) Cell death was determined using Annexin V and 7-AAD. (e) TRIM44^{KD} or TRIM44^{OE} cells were generated using lentivirus-mediated silencing or overexpression, which led to TRIM44 knock down or TRIM44 overexpression. Control cells were generated using appropriate control vectors for knock down or overexpression. (f) TRIM44^{OE} or (g) TRIM44^{KD} were generated in 293T cells. The TRIM44 protein levels were analyzed using immunoblots.

Supplementary Figure 4

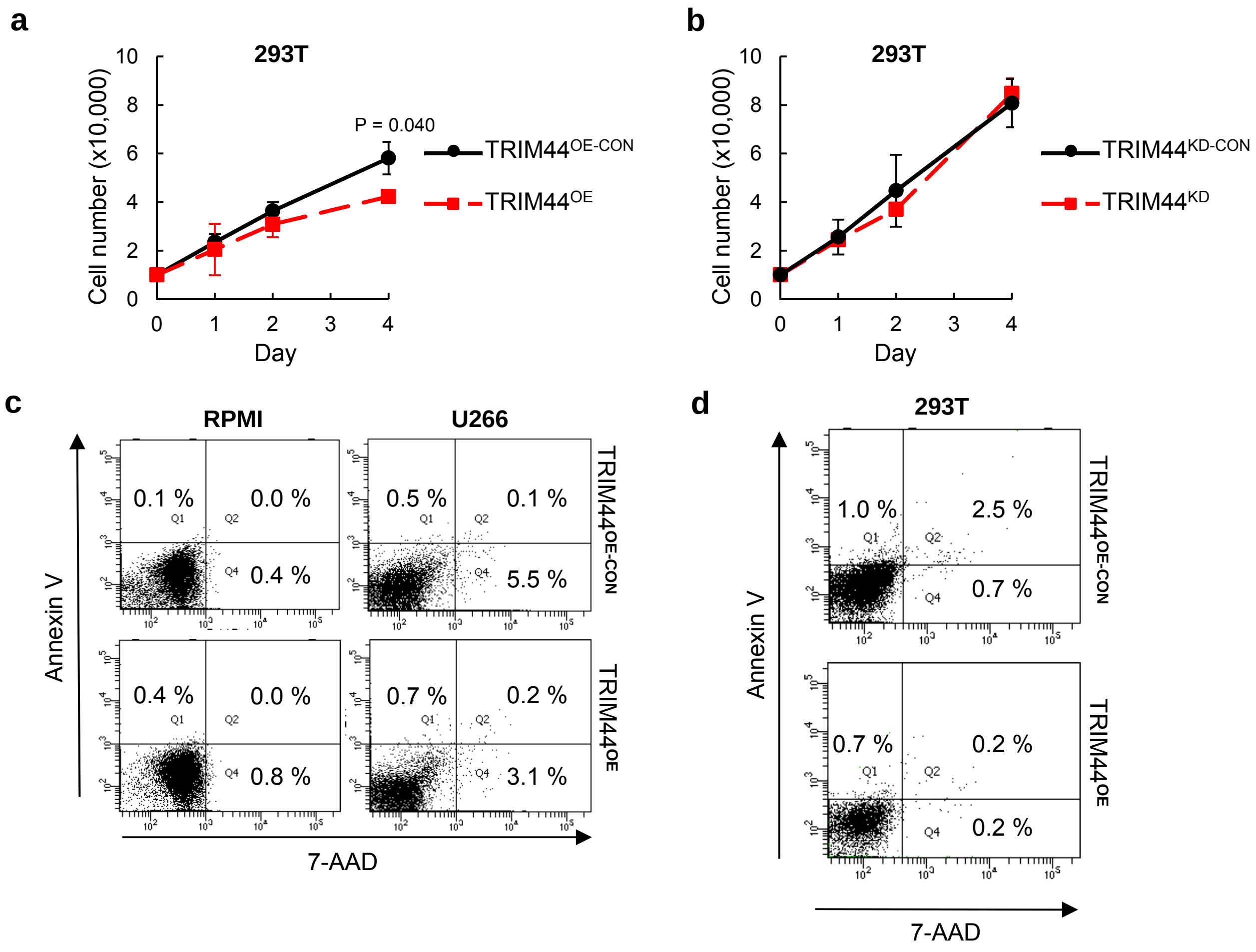

Figure S4. (a & b) TRIM44 overexpression (TRIM44^{OE}) delayed 293T cell proliferation (a), whereas TRIM44^{KD} did not change cell proliferation (b). (c) TRIM44$^{\mathbf{OE\text{-}CON}}$ and TRIM44$^{\mathbf{OE}}$ MM cells display a similar level of apoptotic cell death. The percentage of apoptotic cell death was examined using PE-Annexin V and 7-AAD analyses. (d) TRIM44 overexpression in 293T cells does not increase cell death, which is examined by Annexin V and 7-AAD.

Supplementary Figure 5

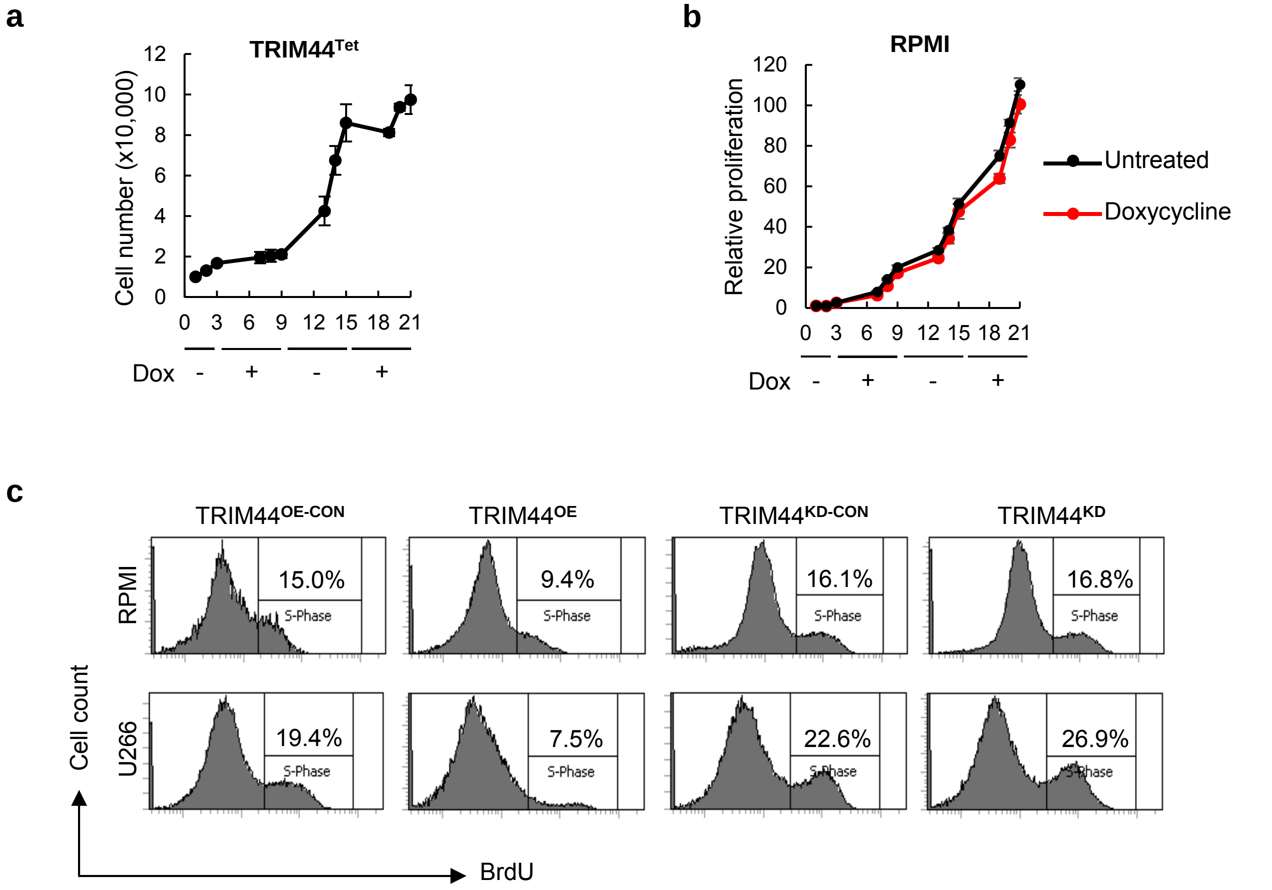

Figure S5. (a) Tet-inducible TRIM44 was generated to monitor effects of TRIM44 expression on MM cell proliferation. RPMI cells expressing inducible TRIM44 were treated 0.5 μg/ml doxycycline for three days (Day 4-6, 16-18) and counted for three days (Day 7-9, 19-21, mark as "+"). Then doxycycline was removed for three days (Day 10-12) , and cell number was counted for three days (Day 13-15, mark as "-"). (b) Doxycycline did not change proliferation of MM cells. RPMI cells were treated with/without 0.5 μg/ml doxycycline for three days (Day 4-6, 16-18) and counted for three days (Day 7-9, 19-21, mark as "+"). Then doxycycline was removed for three days (Day 10-12) , and cell number was counted for three days (Day 13-15, mark as "-"). (c) The percentage of the TRIM44$^{KD\text{-}CON}$, TRIM44^{KD}, TRIM44$^{OE\text{-}CON}$ and TRIM44^{OE} RPMI and U266 cells that were in S-phase was determined using BrdU staining (APC BrdU Flow Kit).

Supplementary Figure 6

a

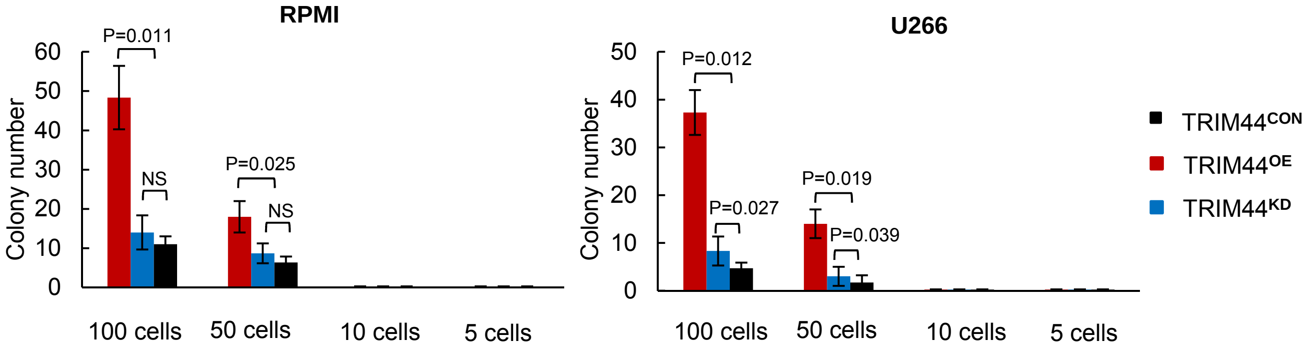

b

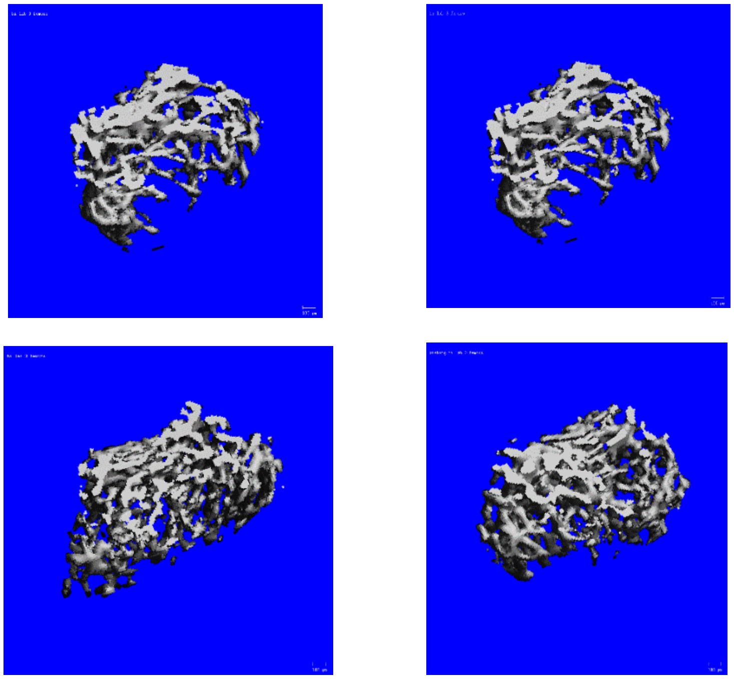

TRIM44^{OE} MM

TRIM44^{KD}TRIM44^{OE} MM

Figure S6. (a) TRIM44^{OE} cells increased colony formation in PHA-LCM methylcellulose medium. (b) 3D analyses of bones (femurs) in mice injected TRIM44^{OE} or TRIM44^{KD} in TRIM44^{OE} (TRIM44^{KD}TRIM44^{OE}) RPMI MM cells (1x10^{6} cells). The femurs were isolated after 4 weeks and 3D bone structure was scanned at the Baylor College of Medicine.

Supplementary Figure 7

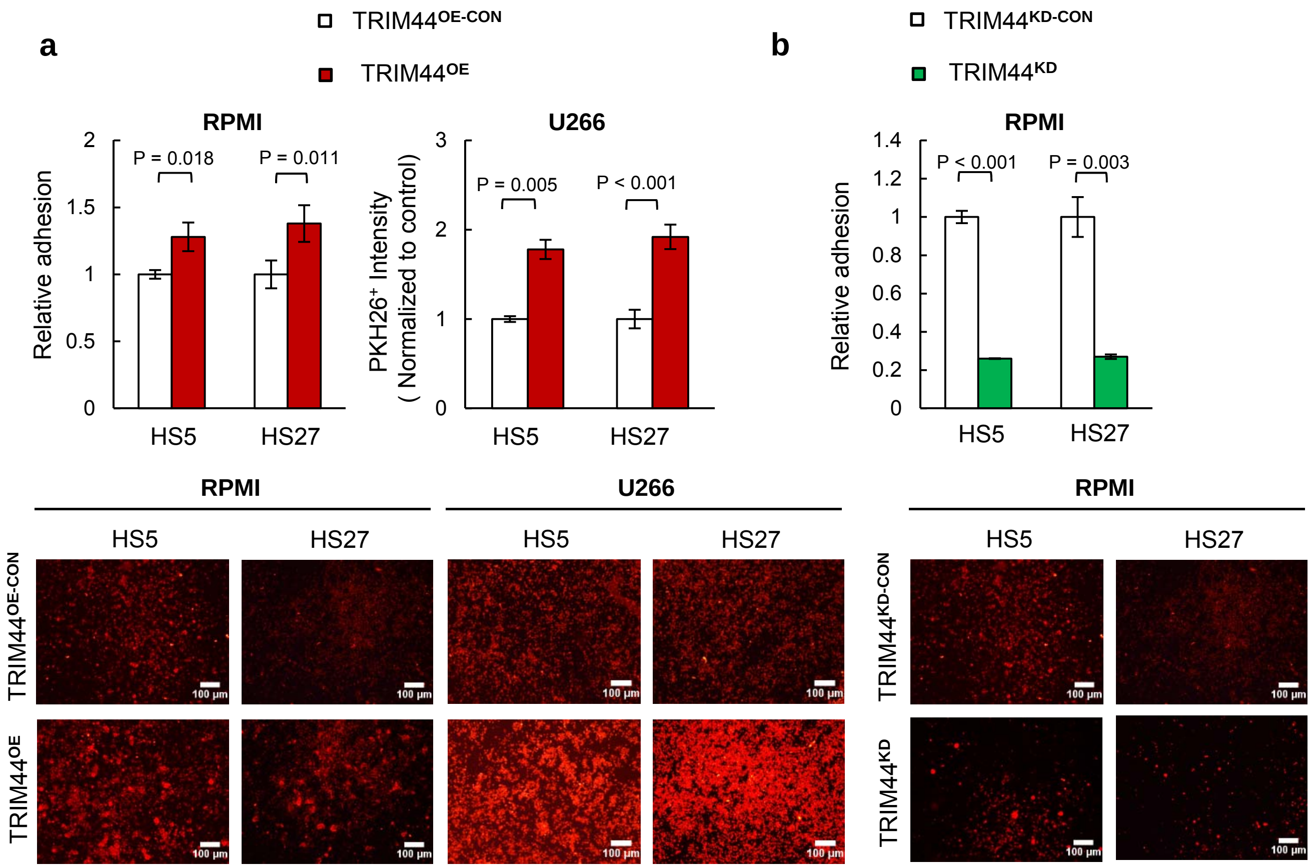

Figure S7. TRIM44 expression in MM cells increases cell adhesion to bone marrow stromal cells. (a) TRIM44 expression (TRIM44^{OE}) increases MM cell adhesion to human stromal cells (HS5 and HS27). PKH26-labelled MM cells (2 x 10^4) were plated on an established monolayer of HS5 or HS27 cells and allowed to adhere for one hour at 37°C. Relative adhesion was determined based on PKH intensity of adhered MM cells. (b) TRIM44 knock down (TRIM44^{KD}) decreases MM cell adhesion to stromal cells. Relative adhesion was determined as described in (a).

Supplementary Figure 7

c

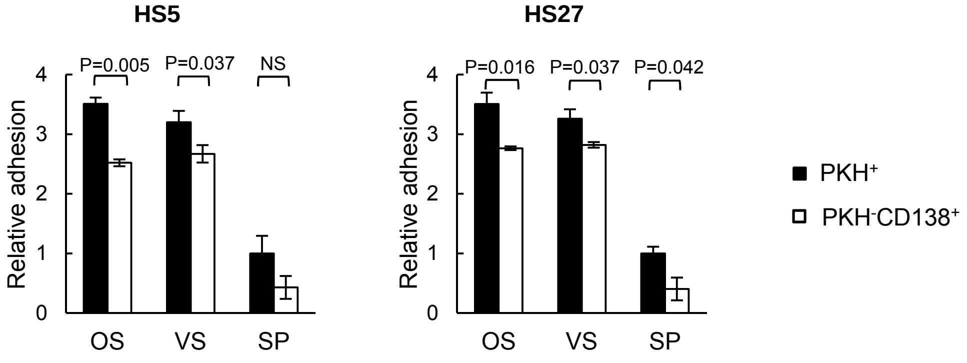

Figure S7. (c) PKH^+ MM cells showed higher adhesion capability. PKH67-labelled RPMI cells were transferred to immunodeficient mice and isolated after 60 hours later. PKH^+ cells and PKH^-CD138^+ cells from mouse BM were isolated using FACS and adhesion assay was performed as described in materials and methods. The statistic significance was calculated and represented as P values.

Supplementary Figure 8

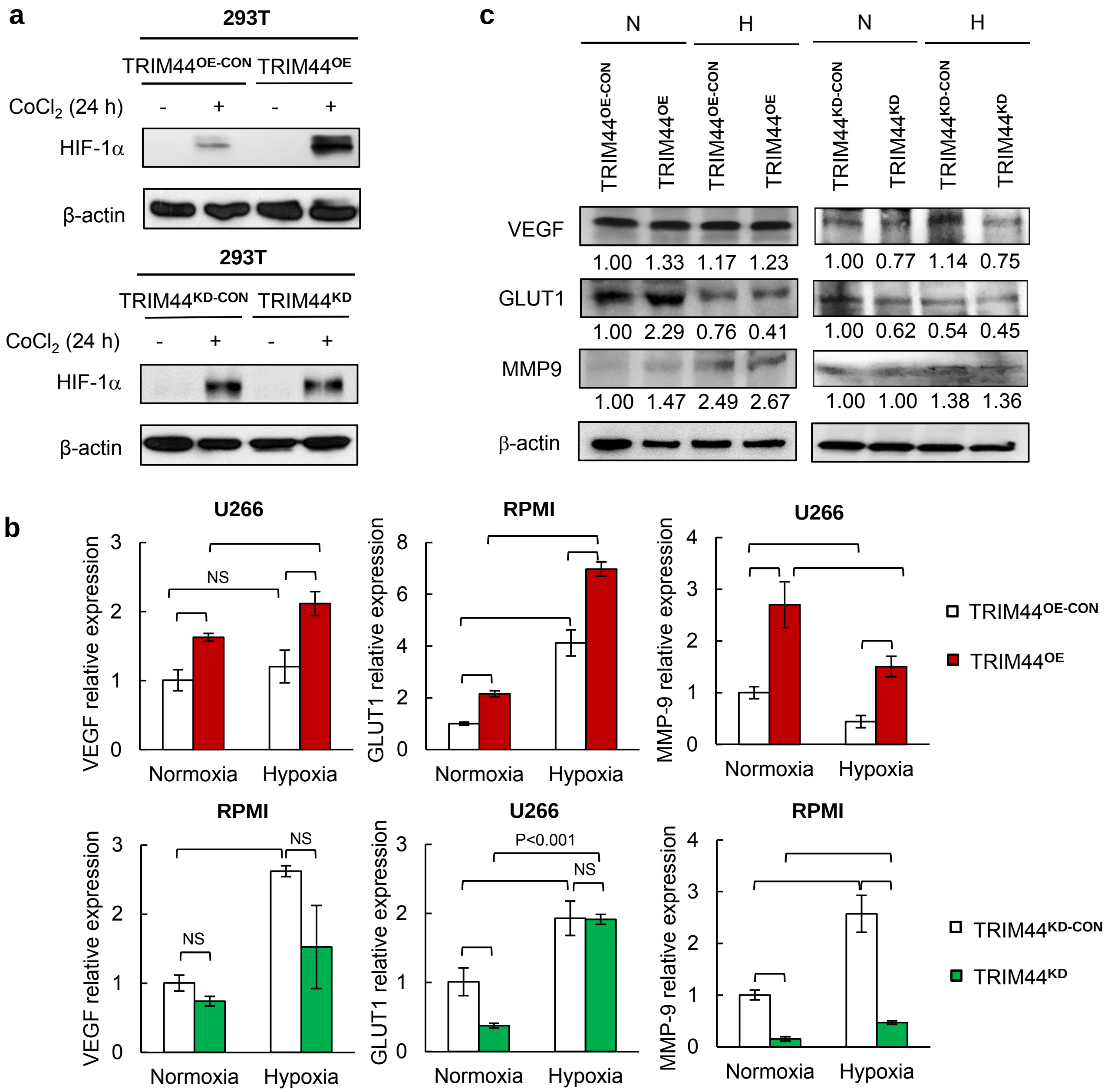

Figure S8. TRIM44 stabilize HIF-1α expression via deubiquitination. (a) A higher level of HIF-1α is induced after $CoCl_2$ treatment (24 hours) in TRIM44^{OE} MM cells. (b & c) TRIM44 expression (TRIM44^{OE}) in U266 cells enhances VEGF, GLUT1, and MMP-9 expression and TRIM44 silencing (TRIM44^{KD}) decreases their expression. U266 cells were incubated in normoxia or hypoxia (1% O_2) for 24 hours. VEGF, GLUT1, and MMP9 expression were determined using real-time PCR (b) and immunoblotting (c). The relative expressions were normalized based on β-actin and GAPDH expression respectively and compared with TRIM44^{CON} cells under normoxia. N: Normoxia; H: Hypoxia.

Supplementary Figure 9

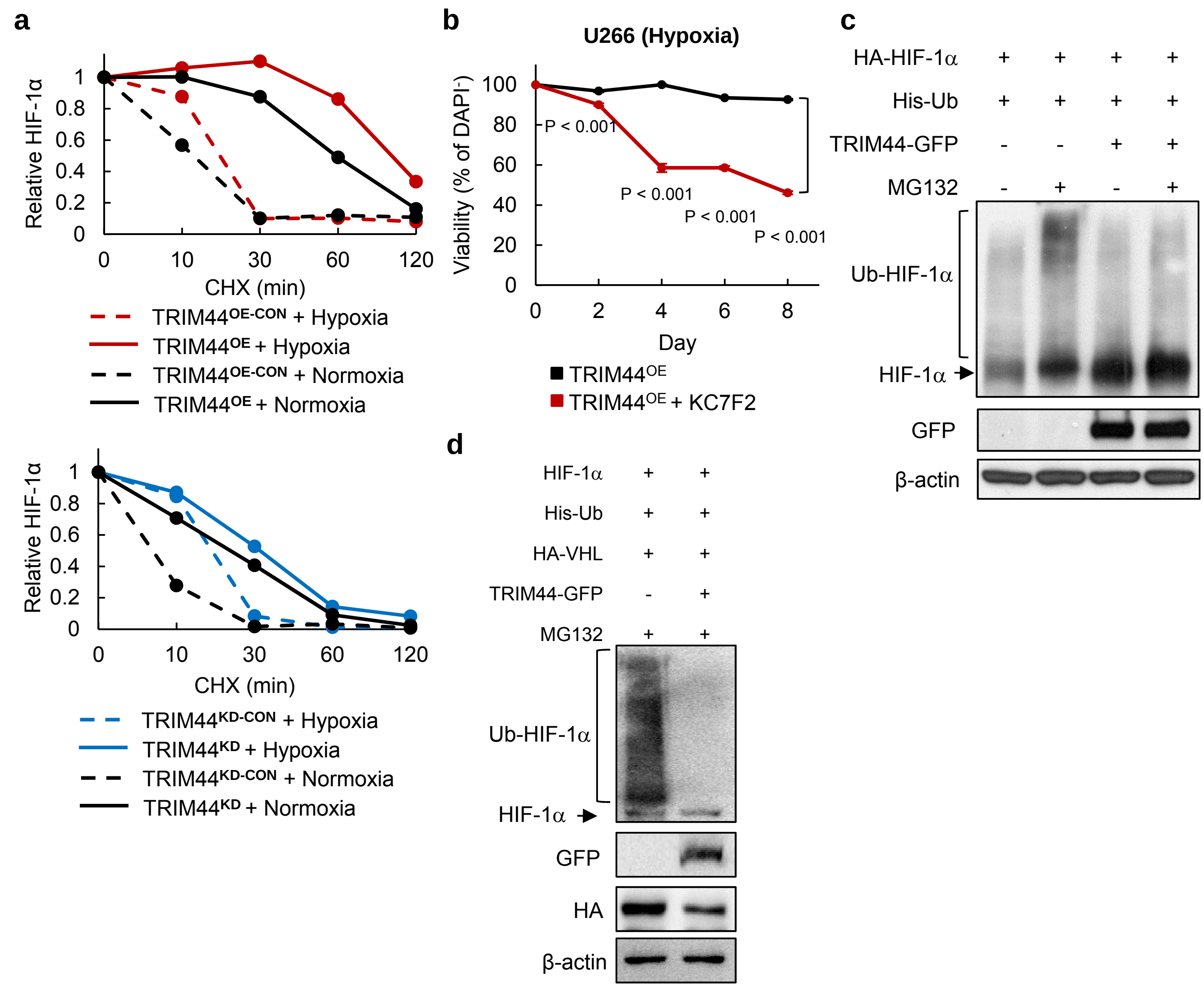

Figure S9. (a) $TRIM44^{OE-CON}$, $TRIM44^{OE}$, $TRIM44^{KD-CON}$ and $TRIM44^{KD}$ RPMI MM cells were incubated in hypoxia conditions for 4 hours. Subsequently, cycloheximide (CHX, 50 mg/ml) was added for 0, 10, 30, 60 and 120 min under normoxia or hypoxia conditions. Quantitation of HIF-1α expression was normalized by β-actin. Relative HIF-1α expression at 0 min was set as 1.00. (b) KC7F2 treatment reduces $TRIM44^{OE}$ viability. $TRIM44^{OE}$ MM cells were grown under hypoxia and treated with KC7F2. Cell viability was monitored through DAPI staining using FACS. (c) Cells (293T) were transfected with HA-HIF-1α, His-Ub, and TRIM44-GFP and were treated with or without MG132 (5 μM, 6 hours). Ubiquitinated HIF-1α (> 120 kD) was then analyzed by immunoblotting. (d) HA-HIF-1α, His-Ub, HA-VHL or TRIM44-GFP transfected 293T cells were treated with MG132, and ubiquitinated HIF-1α was analyzed using immunoblotting.

Supplementary Figure 10

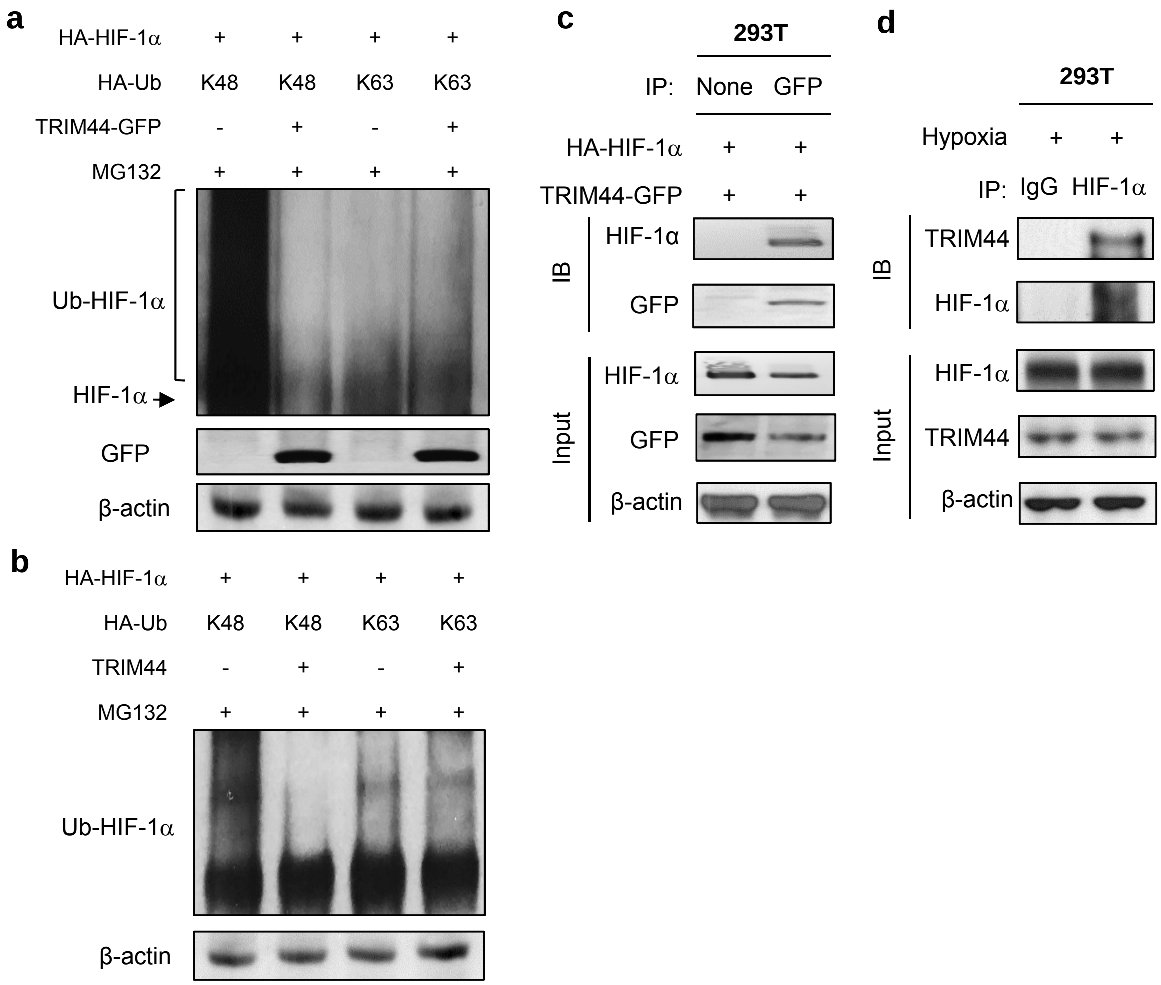

Figure S10. (a) TRIM44 decreases K48-linked ubiquitination of HIF-1α. 293T cells were transfected with various combinations of HA-HIF-1α, HA-Ub-K48, HA-Ub-K63, and TRIM44-GFP. The cells were then treated with MG132 (5 μM, 6 hours). Ubiquitinated HIF-1α (> 120 kD) was measured by immunoblotting. (b) TRIM44 decreases K48-linked ubiquitination of HIF-1α. TRIM44$^{OE\text{-}CON}$ and TRIM44^{OE} MM cells were transfected with a combination of HA-HIF-1α, HA-Ub-K48, or HA-Ub-K63. Cells were treated with MG132 (5 μM) for 6 hours before collection. The level of ubiquitinated HIF-1α (> 120 kD) was measured using immunoblotting. (c) TRIM44 interacts HIF-1α in transiently transfected 293T cells. 293T cells were transfected with HA-HIF-1α and TRIM44-GFP. Cell lysates were immunoprecipitated with anti-GFP and immunoblotting with GFP or HIF-1α. (d) HIF-1α expression was induced by incubating cells in hypoxia (low oxygen condition, 1%. 4 hours). Immunoprecipitation and immunoblotting were performed to determine expression.
