# Supplementary material for: TRIM44 promotes quiescent multiple myeloma cell occupancy and survival in the osteoblastic niche via HIF-1α stabilization
Source: Leukemia. 2018 Aug 8;33(2):469–86. doi: 10.1038/s41375-018-0222-x (PMC6365383; doi:10.1038/s41375-018-0222-x)
Supplement: Supplementary file 2 — Supplemental Table [file 41375_2018_222_MOESM2_ESM.pdf]

Table S1 for Figure 1A

Type of MM	# of MM cells injected	# of CD34+ HSCs injected	Time	# of mouse	Tissue examined
-	-	10^6	60 h	12	BM
NCI	$5x10^5$	10^6	60 h	6	BM
	10^6	10^6	60 h	6	BM
U266	$5x10^5$	10^6	60 h	12	BM
	10^6	10^6	60 h	12	BM
RPMI	$5x10^5$	10^6	60 h	12	BM
	10^6	10^6	60 h	12	BM

Table S2 for Figures 1B & C

Type of MM	# of MM cells injected	# of CD34+ HSCs injected	Time	# of mouse	Tissue examined
-	-	10^6	16 w	6	Blood/BM
RPMI	$5 x 10^5$	10^6	16 w	6	Blood/BM
	10^6	10^6	16 w	6	Blood/BM
	$3 x 10^6$	10^6	16 w	6	Blood/BM

Table S3 for Figure 1D

Type of MM	# of MM cells injected	# of CD34+ HSCs injected	Time	# of mouse	Tissue examined
-	-	10^6	60 h	12	OS/VS
NCI	$5x10^5$	10^6	60 h	6	OS/VS
	10^6	10^6	60 h	6	OS/VS
U266	$5x10^5$	10^6	60 h	9	OS/VS
	10^6	10^6	60 h	9	OS/VS
RPMI	$5x10^5$	10^6	60 h	12	OS/VS
	10^6	10^6	60 h	12	OS/VS

Table S4 for Figure 1E

Type of MM	# of MM cells injected	# of CD34+ HSCs injected	Time	# of mouse	Tissue examined
-	-	10^6	16 w	6	OS/VS
RPMI	5×10^5	10^6	16 w	6	OS/VS
	10^6	10^6	16 w	6	OS/VS
	3×10^6	10^6	16 w	6	OS/VS

Table S5 for Figure 2B

Type of MM	# of MM cells injected	Mouse pretreated	Time	# of mouse	Tissue examined
RPMI8226	10^6	Vehicle	60 h	3	OS / VS
RPMI8226	10^6	AMD3100	60 h	3	OS / VS

Table S6 for Figure 2C

Type of MM	# of MM cells injected	Mouse pretreated	Time	# of mouse	Tissue examined
RPMI8226	10^6	Vehicle	60 h	3	OS / VS
RPMI8226	10^6	PTH	60 h	3	OS / VS
U266	10^6	Vehicle	60 h	3	OS / VS
U266	10^6	PTH	60 h	3	OS / VS

Table S7 for Figure 3A

Type of MM	# of MM cells injected	Time	# of mouse	Tissue examined
RPMI8226 TRIM44$^{OE\text{-}CON}$	10^6	60 h	3	OS / VS
RPMI8226 TRIM44^{OE}	10^6	60 h	3	OS / VS
U266 TRIM44$^{OE\text{-}CON}$	10^6	60 h	3	OS / VS
U266 TRIM44^{OE}	10^6	60 h	3	OS / VS

Table S8 for Figure 3B

Type of MM	# of MM cells injected	Time	# of mouse	Tissue examined
RPMI8226 TRIM44$^{KD\text{-}CON}$	10^6	60 h	3	OS / VS
RPMI8226 TRIM44^{KD}	10^6	60 h	3	OS / VS
U266 TRIM44$^{KD\text{-}CON}$	10^6	60 h	3	OS / VS
U266 TRIM44^{KD}	10^6	60 h	3	OS / VS

Table S9 for Figures 3C & D

Type of MM	# of MM cells injected	Mouse pretreated	Time	# of mouse	Tissue examined
U266 $TRIM44^{OE\text{-}CON}$	10^6	Vehicle	60 h	3	OS / VS
U266 $TRIM44^{OE}$	10^6	Vehicle	60 h	3	OS / VS
U266 $TRIM44^{KD\text{-}CON}$	10^6	Vehicle	60 h	3	OS / VS
U266 $TRIM44^{KD}$	10^6	Vehicle	60 h	3	OS / VS
U266 $TRIM44^{OE\text{-}CON}$	10^6	PTH	60 h	3	OS / VS
U266 $TRIM44^{OE}$	10^6	PTH	60 h	3	OS / VS
U266 $TRIM44^{KD\text{-}CON}$	10^6	PTH	60 h	3	OS / VS
U266 $TRIM44^{KD}$	10^6	PTH	60 h	3	OS / VS
RPMI $TRIM44^{OE\text{-}CON}$	10^6	Vehicle	60 h	3	OS / VS
RPMI $TRIM44^{OE}$	10^6	Vehicle	60 h	3	OS / VS
RPMI $TRIM44^{KD\text{-}CON}$	10^6	Vehicle	60 h	3	OS / VS
RPMI $TRIM44^{KD}$	10^6	Vehicle	60 h	3	OS / VS
RPMI $TRIM44^{OE\text{-}CON}$	10^6	PTH	60 h	3	OS / VS
RPMI $TRIM44^{OE}$	10^6	PTH	60 h	3	OS / VS
RPMI $TRIM44^{KD\text{-}CON}$	10^6	PTH	60 h	3	OS / VS
RPMI $TRIM44^{KD}$	10^6	PTH	60 h	3	OS / VS

Table S10 for Figure 3E

Type of MM	# of MM cells injected	Mouse pretreated	Time	# of mouse	Tissue examined
RPMI8226 TRIM44$^{OE\text{-}CON}$	10^6	Vehicle	60 h	3	OS / VS
RPMI8226 TRIM44$^{OE\text{-}CON}$	10^6	Vehicle	60 h	3	OS / VS
RPMI8226 TRIM44^{OE}	10^6	AMD3100	60 h	3	OS / VS
RPMI8226 TRIM44^{OE}	10^6	AMD3100	60 h	3	OS / VS

Table S11 for Figure 4A

Type of MM	# of MM cells injected	# of CD34+ HSCs injected	Time	# of mouse	Tissue examined
RPMI8226 TRIM44$^{OE\text{-}CON}$	0.5×10^6	10^6	60 h	3	OS / VS
RPMI8226 TRIM44$^{OE\text{-}CON}$	10^6	10^6	60 h	3	OS / VS
RPMI8226 TRIM44^{OE}	0.5×10^6	10^6	60 h	3	OS / VS
RPMI8226 TRIM44^{OE}	10^6	10^6	60 h	3	OS / VS
U266 TRIM44$^{OE\text{-}CON}$	0.5×10^6	10^6	60 h	3	OS / VS
U266 TRIM44$^{OE\text{-}CON}$	10^6	10^6	60 h	3	OS / VS
U266 TRIM44^{OE}	0.5×10^6	10^6	60 h	3	OS / VS
U266 TRIM44^{OE}	10^6	10^6	60 h	3	OS / VS

Table S12 for Figure 4C

Type of MM	# of MM cells injected	# of CD34+ HSCs injected	Time	# of mouse	Tissue examined
RPMI8226 TRIM44^{KD-CON}	10^6	10^6	60 h	3	OS / VS
RPMI8226 TRIM44^{KD}	10^6	10^6	60 h	3	OS / VS
U266 TRIM44^{KD-CON}	10^6	10^6	60 h	3	OS / VS
U266 TRIM44^{KD}	10^6	10^6	60 h	3	OS / VS

Table S13 for Figure 5A

Type of MM	# of MM cells injected	# of CD34+ HSCs injected	Time	# of mouse	Tissue examined
-	-	10^6	16 w	3	OS / VS
RPMI8226 TRIM44^{OE-CON}	$0.5x10^6$	10^6	16 w	3	OS / VS
RPMI8226 TRIM44^{OE-CON}	10^6	10^6	16 w	3	OS / VS
RPMI8226 TRIM44^{OE-CON}	$3x10^6$	10^6	16 w	3	OS / VS
RPMI8226 TRIM44^{OE}	$0.5x10^6$	10^6	16 w	3	OS / VS
RPMI8226 TRIM44^{OE}	10^6	10^6	16 w	3	OS / VS
RPMI8226 TRIM44^{OE}	$3x10^6$	10^6	16 w	3	OS / VS

Table S14. Primer sequences for real time-PCR reactions used in the article.

Gene	Sense	Anti-sense
**Real-time PCR**		
TRIM44	AGGCAGCTCATCTGTGTCCT	TGCTCCTCATCAGCAATCAC
VEGF	GGAGTGTGTGCCCACCGAGGAGTCCAAC	GGTTCCCGAAACCCTGAGGGAGGCT
GLUT1	CTACAACACTGGAGTCATCAATGC	GGCCAGCAGGTTCATCATCAGCAT
MMP9	GAGTGGCAGGGGGAAGATGC	CCTCAGGGCACTGCAGGATG
ACTB	GGACTTCGAGCAAGAGATGG	AGCACTGTGTTGGCGTACAG
