# Supplementary material for: TRIM44 promotes quiescent multiple myeloma cell occupancy and survival in the osteoblastic niche via HIF-1α stabilization
Source: Leukemia. 2018 Aug 8;33(2):469–86. doi: 10.1038/s41375-018-0222-x (PMC6365383; doi:10.1038/s41375-018-0222-x)
Supplement: Supplementary file 3 — Supplemental Methods [file 41375_2018_222_MOESM3_ESM.pdf]

Materials and methods

Plasmids and chemicals

Plasmids HA-HIF1α-pcDNA3 (#18949), HA-VHL-pRc/CMV (#19999), pRL-SV40P (#27163), pRK5-HA-Ubiquitin-K48 (#17605), pRK5-HA-Ubiquitin-K63 (#17606), and mCherry2-N1 (#54517) were obtained from Addgene. Reporter pGL2-basic vector was purchased from Promega. TRIM44-GFP was constructed by cloning TRIM44 coding sequence from HEK293T cDNA and inserting into pEGFP-N1. Lentiviral vectors for open reading frame of human TRIM44 and shRNA against human TRIM44 were purchased from GE Dharmacon Open Biosystems. Cobalt(II) chloride and cycloheximide were from Sigma-Aldrich, MG132 from Selleckchem. Doxorubicin and etoposide were acquired from the MD Anderson Cancer Center drug repository for cell cytotoxicity studies.

Animals

Animal experiments were conducted in accordance with accepted standards of humane animal care and approved by the Animal Care and Use Committees at the University of Texas-Health Science Center at Houston. All animal experiments were performed using age-matched male and female mouse littermates. All NOD/SCID mice (NOD.CB17-Prkdcscid/J, Jackson Lab) were 4-8 week old. After the selection of the mice, to remove a possible biases, all the treatment were randomly assigned. All the bone analyses were performed in the Baylor College of Medicine in a blind manner. Detailed animal numbers used for

the experiments are described in Supplemental Tables. Sample numbers were estimated to achieve significance.

MM, MGUS and normal BM sections

Bone sections were obtained from MD Anderson Cancer Center. Those samples at MD Anderson were originated from the study was approved by the MD Anderson Cancer Center Institutional Review Board (PA13-0505) after informed consent. All study was originally performed in accordance with the Declaration of Helsinki. Biopsies for histological examination were chosen from archived patient samples of paraffin-embedded tissue from the Department of Hematopathology at MD Anderson Cancer Center. All the samples were analyzed in a blind manner.

Cell culture

The human MM cell lines NCI-H929, RPMI 8226, and U266 were obtained from the American Type Culture Collection (Manassas, VA). These cell lines are performed a routine mycoplasma contamination test. All cell lines were recently authenticated using short Tandem Repeat (STR) DNA profiling, also known as DNA fingerprinting. HS5 and HS27 BM stromal cells were kind gifts from Dr. B. Torok-Storb (Fred Hutchinson Cancer Research Center). Cells were maintained in RPMI1640 medium supplemented with 10% fetal bovine serum (FBS) under 5% CO_2 condition at 37°C. For hypoxia treatment, cells were grown under 1% O_2 and 5% CO_2 condition at 37°C for indicated time or treated with 200 μM $CoCl_2$ at

37°C for 24 h. However, no cells were extensively cultured more than 1.5 months due to a possible genetic modification occuring during culture. Gene targeted cells were verified using immunoblots after generation and frozen. After 1.5 month later new cell stocks were introduced to minimize genetic modification. All cells verified prior to experiments.

Antibodies

The following antibodies were purchased for the experiments. anti-CD138 (BD Pharmingen, Cat# 552723 or eBioscience, Cat# 17-1389-41), anti-CD34 (BD Pharmingen, Cat# 555824), anti-CD45 (BD Pharmingen, Cat# 555483), anti-HIF-1a (Novus Biologicals, Cat# NB100-134), anti-GFP (Proteintech; Cat# 66002-1-Ig), anti-TRIM44 (proteintech; Cat# 11511-1-AP), anti-GFP (Abcam; Cat# ab5450), anti-β-actin (Santa Cruz Biotechnology; Cat# sc-47778).

Isolation of osteoblastic niche and vascular niche from bones of NOD/SCID mice and FACS analysis

Very detailed methods of isolating MM cells from osteoblastic niche and vascular niche are described in our previously manuscript: Chen et al, *Blood*, 2014 (1).

RNA extraction and RT- PCR

Total RNA was extracted using Direct-zol™ RNA MiniPrep (Zymo Research) and cDNA synthesized using random hexamers and ImProm-II™ Reverse Transcription System (Promega). The synthesized cDNA was 1:10 diluted in nuclease-free water for PCR amplification. Quantitative real-time PCR was performed using SYBR Green MasterMix Plus (Thermo Scientific) on ABI-7900 (Applied Biosystems) with initiating temperature 95°C for 10 min, followed by 40 cycles of 15 s at 95°C, 30 s at 60°C, and 30 s at 72°C. *ACTB* expression served as internal control to normalize *TRIM44* expression. For semi-quantitative RT-PCR, the diluted cDNA as template was amplified with Qiagen HotStar Taq (Qiagen) for 35 cycles on Bio-Rad thermocycler (Bio-Rad). The PCR products were determined by using agarose electrophoresis. *GAPDH* expression served as the internal control. The primer sequences were described in Supplementary Table 14.

Lentivirus production and transduction

HEK293FT cells were transfected with either shRNA against human TRIM44, open reading frame of human TRIM44, or a lentivirus plasmid containing GFP, coupled with pMD2.G and psPAX2 by using calcium phosphate precipitation. Lentiviruses were collected 48 and 72 h post-transfection and concentrated via ultracentrifugation. For transduction, MM cells and HEK293T cells were treated with 5 ug/mL polybrene and transduced with lentivirus. Medium containing lentivirus particles was replaced with regular medium after 24 h. At 96 h post-transduction, cells were sorted through BD FACS Aria II system (BD

Biosciences) or selected with 5 ug/mL blasticidin S or 1 ug/mL puromycin for 7 days, to generate stable cell lines.

Cell proliferation assay

HEK293T cells or MM cells were incubated under 20 % or 1 % O_2 condition at 37°C. At indicated time, MM cell number was counted, and HEK293T cell number was determined by using CellTiter-Blue® (Promega). After adding CellTiter-Blue®, the 560Ex/590Em fluorescent signal was measured using Infinite®M1000 plate reader (Tecan) equipped with SoftMax Pro software (Molecular Devices).

Cell cycle analysis

MM cells were starved with RPMI medium plus 1%FBS for 72 h and then replenished with RPMI medium plus 10%FBS for 72 h. Cells were suspended and fixed in ice-cold 70% ethanol for 1 h, treated with 100 μg/ml RNase A, and stained with 50 μg/ml propidium iodide for 20 min. Cell cycle analysis was performed using LSR-II flow cytometer.

Apoptosis assay

Cells were stained with PE-Annexin V and 7-AAD (BD Biosciences) and detected through LSR-II flow cytometer. Apoptosis index were analyzed by using FACS Diva software (BD Biosciences).

Cell adhesion assay

HS5 or HS27a human stromal cells were seeded in 96-well plate to form monolayer overnight. MM cells were stained with PKH and plated onto pre-established HS5 or HS27a monolayer for 1 h at 37°C in 5% CO_2. After washing out unattached MM cells with PBS, PKH intensity was determined using Infinite®M1000 (Tecan) fluorescence plate reader and photographed using Olympus IX70 fluorescence microscope.

Immunoprecipitation and immunoblotting

For immunoprecipitation, cells were lysed in RIPA buffer coupled with protease inhibitor (Sigma) and incubated with anti-HIF-1α (Novus Biologicals) or anti-GFP (Proteintech) together with protein A/G plus-agarose immunoprecipitation reagent (Santa Cruz Biotechnology) at 4°C overnight. After four washes with RIPA buffer, the immunoprecipitates were eluted with Laemmli sample buffer (Bio-Rad) and analyzed using immunoblotting. For immunoblotting, cells were collected and lysed in Laemmli sample buffer. Proteins were separated by SDS-PAGE and transferred onto PVDF membrane. Anti-HIF-1α, anti-TRIM44, anti-GFP, and anti-β-actin antibodies were used to probe the membrane, followed by

blotting with ECL reagent. β-actin expression was set as internal control to normalize target protein expression.

Statistical analysis

To ensure robust and unbiased results, all results were repeated by multiple times (more than 3 times). All experiments were performed in triplicate or more. Statistical analyses were conducted using GraphPad Prism version 5 (GraphPad Software, Inc) using student's 2-tailed t-test. Criteria for statistical significance is shown in each figure with p values compared to controls. All data are presented as mean ± SEM. Comparison of two groups was performed using unpaired t test. Paired t test was used for comparison of repeated-measures in the same group.

1. Chen Z, Orlowski RZ, Wang M, Kwak L, McCarty N. Osteoblastic niche supports the growth of quiescent multiple myeloma cells. Blood 2014;123:2204-8.
